# Novel H16N3 avian influenza viruses isolated from migratory gulls in China in 2023

**DOI:** 10.3389/fmicb.2024.1543338

**Published:** 2025-01-24

**Authors:** Peng Peng, Jinyan Shen, Wenjun Shi, Jing Guo, Mengjing Wang, Wenxi Li, Zhiqin Yue, Xiaohong Sun, Mengdi Guan, Lili Liu, Hongke Xu, Yujiao Xie, Anran Ren, Mingfeng Liu, Wenqiang Liu, Zhibin Zhang, Zhishu Xiao, Xuyong Li

**Affiliations:** ^1^State Key Laboratory of Integrated Management of Pest Insects and Rodents, Institute of Zoology, Chinese Academy of Sciences, Beijing, China; ^2^University of Chinese Academy of Sciences, Beijing, China; ^3^Biological Disaster Control and Prevention Center, National Forestry and Grassland Administration, Shenyang, China; ^4^College of Agriculture and Biology, Liaocheng University, Liaocheng, China; ^5^Technology Center of Qingdao Customs, Qingdao, China

**Keywords:** avian influenza viruses, H16N3, H5N1, migratory birds, gulls

## Abstract

As a rare subtype of avian influenza virus, H16 viruses are predominant in gulls but rarely found in domestic birds. The low prevalence of H16 viruses has limited our understanding of their epidemiology and evolutionary dynamics. In this study, we isolated three novel H16N3 viruses from migratory gulls in East Asian–Australasian Flyway in eastern China in 2023, which are significantly different from previously identified isolates. To fully understand the epidemiology and genetics characteristics of the global H16 viruses, we compared the host divergence of several rare subtypes and determined that the H13 and H16 subtypes were predominantly pooled into different species of gulls by sharing their internal genes, whereas the waterfowl of *Anatidae* served as the primary natural reservoirs of the H8, H11, H12, H14, and H15 subtypes. Detailed phylogenetic analysis revealed the evolutionary divergence of globally circulating H16 viruses and their frequent gene reassortment. Furthermore, the gull origin H13 and H16 viruses collectively served as gene donors for the newly emerged highly pathogenic clade 2.3.4.4b H5N1 viruses because the H13/H16-like PA, NP, and NS genes have been introduced into circulating H5N1 viruses since May 2022 in Europe. To date, the H5N1 reassortants containing the H13/H16-like gene segments have been detected in wild and domestic birds and resulted in mammal and human infections. These results improve our knowledge of the ecology and genetics of H16 viruses and emphasize the need for surveillance to monitor the emergence of novel avian influenza viruses in migratory birds.

## 1 Introduction

Influenza A viruses are members of the Orthomyxoviridae family with eight single-negative RNA segments. Influenza A viruses have been identified as having 19 hemagglutinin (HA) gene subtypes and 11 neuraminidase (NA) subtypes according to their genetic and antigenic differences (Karakus et al., 2024; Tong et al., 2013; Tong et al., 2012). Avian influenza viruses (AIVs), belonging to influenza A viruses, are widespread in both wild migratory birds and domestic poultry. The emergence and circulation of novel AIVs in recent years have posed continued threats to bird populations and public health (Kumari et al., 2023; Shi et al., 2023).

Migratory birds such as shorebirds (*Charadriiformes*) and waterfowl (*Anseriformes*), are considered natural reservoirs of AIVs and play a key role in the ecology and global dissemination of AIVs (Bi et al., 2024; Shi et al., 2023; Gass et al., 2022; van Dijk et al., 2018). From 2020 to date, the clade 2.3.4.4b H5 highly pathogenic viruses, including the initial H5N8 viruses and the descendant H5N6 and H5N1 viruses, have spread rapidly from Europe to the rest of the world, even to Antarctica, following the migration of wild birds (Cui et al., 2022; Gu et al., 2022; Cui et al., 2020; Lisovski et al., 2024; Banyard et al., 2024). The global dissemination of H5N8, H5N6 and H5N1 viruses has resulted in fatal infections in wild waterfowl, shorebirds, domestic birds, and mammals and is responsible for severe economic losses and public health threats (Peacock et al., 2024; Vergne et al., 2024). The H7N9 viruses that emerged in 2013 in China partially originated from wild bird H7 viruses (HA and NA genes) via reassortment with chicken H9N2 viruses and caused five waves of human infection (Shi et al., 2013; Hou et al., 2024). The occasional spillover of viruses from wild birds to humans, such as the first report of human infection with the H7N4 virus in China in 2018, further emphasized their potential public risk (Tong et al., 2018). The recently reported H19 AIVs detected from gulls revealed that the divergence of AIVs, especially the rare subtypes of AIVs, in different species of migratory birds may be underestimated (Karakus et al., 2024). Active avian influenza surveillance in migratory birds and timely identification of novel viral strains will aid in the early detection of viruses with potential risk (Parums, 2023).

H16 viruses, one of the HA subtypes of AIVs, have been understudied because of their low prevalence in birds, resulting in a limited understanding of their epidemiology, genetics and biological characteristics (Li et al., 2020; Lindh et al., 2017). To date, H16 AIV has been only known to wild birds and has not been found to spill over into chickens or mammals. Gulls are considered as the primary natural reservoir of H16 viruses, as most of these identified H16 viruses are detected in different species of gulls (Wang et al., 2022). Unlike viruses that are commonly detected in domestic birds, the H16N3 virus has not adapted to replicate in domestic birds (ducks and chickens) or mice (Wang et al., 2022; Fereidouni et al., 2014; Tønnessen et al., 2011). However, owing to the rare presence of H16 viruses in birds and the limited number of genetic studies, the evolutionary landscape of H16 viruses has been less investigated. In this study, we isolated three novel H16N3 viruses from migratory gulls in China in 2023 and systematically characterized their genetic evolution in birds.

## 2 Materials and methods

### 2.1 Sample collection

Fecal samples were collected from gulls that resided in the Yellow River Delta (YRD) wetland in Shandong Province, China. The gull populations at the habitat were first confirmed by binoculars and photoed by telephoto lens or drones. The single dropping samples were collected in 2 mL centrifuge tubes containing 1 mL of PBS with penicillin and streptomycin and then transferred to a −80°C freezer for storage.

### 2.2 Virus identification and isolation

The methods used for the identification and isolation of AIV from the fecal samples have been described previously (Wang et al., 2023). Briefly, the samples were centrifuged at 12000 rpm for 5 min and then inoculated into chicken embryo eggs for 48 h. The allantoic fluid was tested via a hemagglutination assay in which 1% chicken red blood cells (RBCs) were used. The RNA of the positive samples was extracted and reverse transcribed to cDNA via a reverse transcription kit (Vazyme, China) with a 12 bp primer (5′-AGCRAAAGCAGG-3′). The segments of the virus were then amplified via PCR via gene segment-specific primers and sequenced on an Applied Biosystems DNA Analyzer (3500xL Genetic Analyzer, United States) according to the manufacturer’s instructions. Sequence data of the viruses were compiled via the SeqMan program (DNASTAR, Madison, WI, United States).

### 2.3 Sequence data acquisition

The HA sequences of all the available H8, H11, H12, H13, H14, H15, H16, and HxN3 viruses detected globally were retrieved from the GenBank and GISAID databases. The sequences of each subtype were first imported into MEGA 7.0 to remove the duplicated sequences, and the number of each subtype in different animal hosts was categorized (the hosts were classified according to *family*) to compare the host divergence of different subtypes. The number of different subtype combinations of H16Nx and HxN3 viruses was categorized and summarized to compare the dominant subtypes of H16Nx (N1-N9) and HxN3 (H1–H16). The representative strains were selected according to their isolation time and region, host species, and the similar identity to construct the phylogenetic tree. The sequence data from GenBank and GISAID were updated to December 18, 2024.

### 2.4 Molecular analysis

The complete sequences (ORF) of global H16 subtype were retrieved from GISAID and GenBank databases, the numbers of each segment were summarized by removing the duplicated data. The sequences were imported to MEGA 7, and the key amino acid substitutions that contribute to receptor binding specificity, replication, pathogenicity and transmission of AIVs in chickens and mammals were summarized and analyzed.

### 2.5 Phylogenetic analysis

For genetic comparison, reference sequences of each gene segment were retrieved from the GISAID and GenBank databases. Phylogenetic analysis of the H16N3 viral gene segments was then performed by constructing phylogenetic trees on the basis of the HA gene, the NA gene and internal genes. Multiple sequence alignment was performed via MAFFT software (v7.505) and then analyzed via MEGA 7. Time-scaled phylogenetic trees with molecular clocks were inferred via BEAST (v1.10.4). The GTR + F + G4 nucleotide substitution model and the relaxed clock log normal clock model were used, and the Coalescent constant population was set as the tree prior. Stationarity and mixing were investigated using Tracer (v1.7.1), making sure that effective sample sizes for the continuous parameters were greater than 200. The maximum clade credibility tree with median node heights was constructed after a burn-in of the beginning states. Phylogenetic trees were visualized via the tvBOT online service^1^.

## 3 Results

### 3.1 Isolation of three H16N3 viruses from migratory gulls

To monitor the circulation of AIV in migratory birds, we carried out continuous viral surveillance in the East Asian–Australasia Flyway (EAAF) of eastern China since 2017 (Supplementary Figure S1). In the autumn migration of 2023, we isolated three H16N3 viruses from 1,342 fecal samples of the gulls in the YRD wetland (Supplementary Table S1 and Supplementary Figure S1). We summarized the H16 viruses previously reported in China and found that only three H16N3 viruses were isolated from gulls in China (Table 1). The two 2018 H16N3 viruses were isolated from great black-headed gulls in the Ningxia Hui Autonomous Region, which is located in the Central Asia flyway (Li et al., 2020), and the other 2021 H16N3 virus reported in our previous study was isolated from gulls in the YRD wetland in Shandong Province (Supplementary Figure S1) (Wang et al., 2022). These results indicate the low prevalence of H16 viruses in migratory birds in China.

**Table 1:** All the available H16 viruses isolated in China deposited in public databases.

NO.	Isolate name	Subtype	Location	Host	Isolate ID	Collection date
1	A/great black-headed gull/Ningxia/1/2018(H16N3)	H16N3	Ningxia Hui Autonomous Region	*Larus marinus*	EPI_ISL_393957	2018-10-23
2	A/great black-headed gull/Ningxia/2/2018(H16N3)	H16N3	Ningxia Hui Autonomous Region	*Larus marinus*	EPI_ISL_393962	2018-10-23
3	A/gull/Shandong/W1359/2021(H16N3)	H16N3	Shandong	Gull	EPI_ISL_15052970	2021-10-10
4^a^	A/gull/Shandong/W4786/2023(H16N3)	H16N3	Shandong	Gull	EPI_ISL_19491465	2023-10-24
5^a^	A/gull/Shandong/W4789/2023(H16N3)	H16N3	Shandong	Gull	EPI_ISL_19492902	2023-10-24
6^a^	A/gull/Shandong/W4807/2023(H16N3)	H16N3	Shandong	Gull	EPI_ISL_19493205	2023-10-24

^aThe H16N3 virus detected in this study. The sequences information of these H16 viruses are available from GISAID.^

### 3.2 Comparison of the host reservoirs of H16 viruses and other rare subtypes

**Figure 1:**
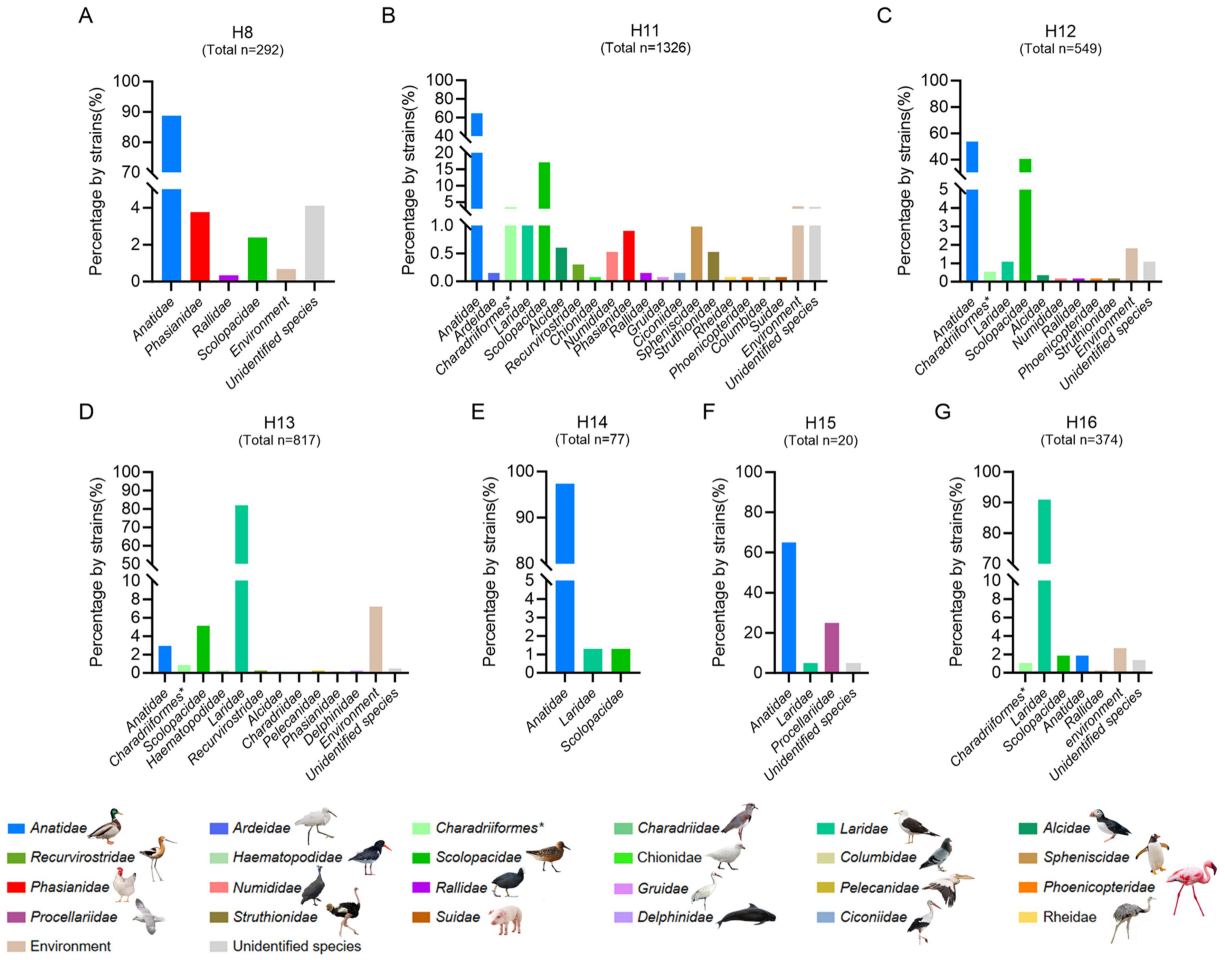
Host reservoirs of H16 and other rare subtype viruses worldwide. **(A)** H8; **(B)** H11; **(C)** H12; **(D)** H13; **(E)** H14; **(F)** H15; **(G)** H16. The viral information of these subtypes, including complete and partial HA sequences was obtained from GenBank and GISAID. The duplicate sequences were removed. The data was updated to December 18, 2024. The host species of each *family* were classified and summarized. *, indicates the *order* of the birds.

Unlike the influenza A viruses, which can be frequently detected in a wide range of birds or mammalian hosts, the subtypes of H8 and H11–H16 are primarily pooled in migratory birds and rarely be detected and reported in domestic birds and mammals (Wang et al., 2022; Wang et al., 2024; Zhang et al., 2024; Shen et al., 2024; Siegers et al., 2024; Ortiz et al., 2023; Sivay et al., 2013). Although the H2 and H10 subtypes have similar strain number with H11 subtype, they were detected in wild birds, poultry, mammals and humans, and were not considered as rare subtypes in this study (Supplementary Figure S2). To better understand the divergence of the natural reservoirs of H16 viruses and other rare subtypes, we summarized the host species of the viruses, including the H8, H11, H12, H13, H14, H15, and H16 subtypes, according to the available viral information in GISAID and GenBank (Figure 1 and Supplementary Table S2). To date, 88.70% of H8 strains (*n* = 292), 97.40% of H14 strains (*n* = 77) and 65.00% of H15 strains (*n* = 20) have been detected in birds of *Anatidae* (Figure 1), whereas 64.63 and 17.12% of H11 strains (*n* = 1,326) and 53.73 and 40.62% of H12 strains (*n* = 549) were identified in birds of *Anatidae* and *Scolopacidae*, respectively. Interestingly, more than 82% of H13 strains (*n* = 817) and more than 91% of H16 strains (*n* = 374) were detected from different species of gulls (*Laridae*) but not from birds of *Anatidae*. To date, only 24 H13 viruses and seven H16 viruses were identified in *Anatidae* (Figure 1). Notably, none of the H12, H14, H15, or H16 viruses and nearly no H8, H11 or H13, viruses were identified in either chickens or mammals (Figure 1). These results suggest that H13 and H16 share shorebirds of *Laridae* as natural reservoirs, and they were significantly different from other subtypes that are common in waterfowl of *Anatidae*.

### 3.3 H16N3 is the dominant subtype combination of H16 viruses

**Figure 2:**
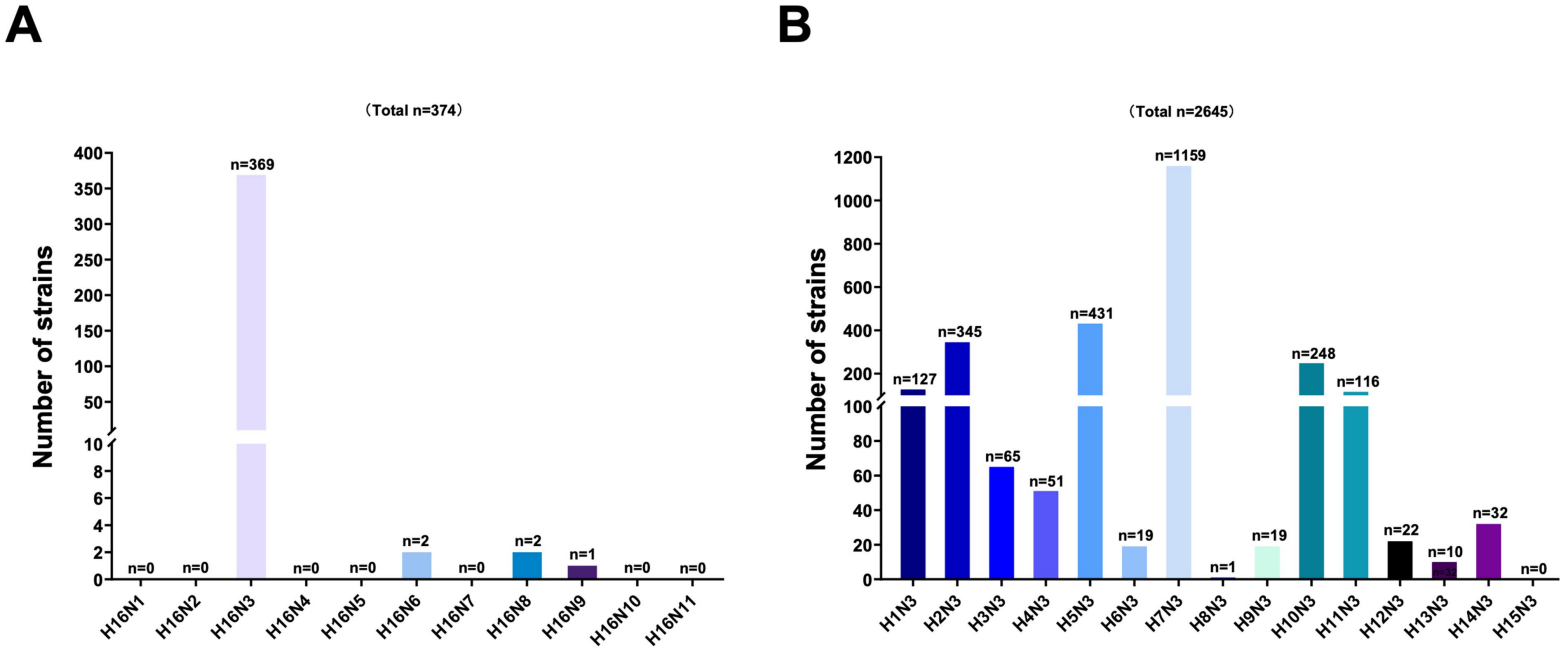
Analysis of the subtype combinations of the HA (H1–H16) and NA (N3) genes. **(A)** Combination bias of the HA (H16) and NA (N1–N9) subtypes. All the global H16 subtypes were retrieved from GenBank and GISAID, and the combinations of HA and NA were classified. **(B)** Combination bias of the HA (H1–H16) and NA (N3) subtypes. The N3 subtypes of AIVs in the databases were classified according to each HA (H1–H16) and N3 combination.

To better understand the viral subtype combinations of the HA (H16) and NA genes, we categorized the H16Nx (N1–N9) strains retrieved from the GISAID and GenBank databases. To date, only 374 H16 strains, including H16N3, H16N6, H16N8, and H16N9, have been identified from birds worldwide. However, the H16N3 (*n* = 369) viruses accounted for the dominance of the known H16 viruses (Figure 2A). Additionally, the NA (N3) genes primarily combined with the H7, H5, H16, and H2 genes, as 38.45% (*n* = 1,159), 14.30% (*n* = 431), 12.24% (*n* = 369) and 11.45% (*n* = 345) of the identified HxN3 (H1-H16) subtypes were H7N3, H5N3, H16N3, and H2N3 viruses, respectively (Figure 2B). These results indicate that H16N3 is the dominant subtype combination of circulated H16 viruses worldwide.

### 3.4 Molecular characteristics of the H16N3 viruses

The receptor binding specificity, replication, virulence and transmissibility of AIVs are highly related to key amino acid substitutions in the segmented viral proteins. We then analyzed amino acid substitutions according to the available full-length genome sequences of all the H16 viruses retrieved from the GISAID and GenBank databases. The duplicated data and the partial sequences were removed and the sequences containing the ORF were summarized for analysis. The consensus amino acid substitutions 183H, 190T, and 226Q at the receptor binding sites in HA were found in the circulated H16 viruses to support their preferential binding specificity to avian-type receptors. Interestingly, the amino acid substitution 228S, which contributes to the increased receptor binding ability of the H3 and H5N1 viruses to human α2-6 sialosides, is found in the HA proteins of all H16 strains (Table 2) (Vines et al., 1998; Stevens et al., 2008; Gao et al., 2009). Most H16 viruses possess the conserved amino acid motif INER/GLF at the cleavage site in HA, which is a typical characteristic of viruses with low pathogenicity in chickens (Table 2). Although the classical amino acid substitutions E627K or D701N in PB2 were not observed in all of the identified H16 viruses, several amino acid substitutions that have been reported to increase replication, virulence or transmissibility in mammals were observed in all or most H16 viruses, including R207K and H436Y in PB1, V41I in NP, N30D and T215A in M1, and V149A in NS1 (Table 2) (Zhu et al., 2015; Guo et al., 2022; Hulse-Post et al., 2007; Li et al., 2006).

**Table 2:** Molecular characteristics of the global H16 viruses.

Segment	HA											PB2		PB1		NP		M1		NS1
Position	Cleavage site					183	190			226	228	627	701	207	436	41		30	215	149
Amino acid	INER/GLF	IVER/GLF	IVDR/GLF	IGER/GLF	ISER/GLF	H	T	V	I	Q	S	E	D	K	Y	I	V	D	A	A
Number*^a^*	230/355	48/355	37/355	15/355	13/355	355/355	345/355	9/355	1/355	355/355	355/355	330/330	330/330	331/331	331/331	322/334	11/334	340/341	341/341	337/337
Viruses in this study	IVER/GLF					H	T			Q	S	E	D	K	Y	I	D		A	A

^aNumber of the segments that contain the ORF sequences.^

### 3.5 Genetic and phylogenetic analyses of the H16N3 viruses

**Figure 3:**
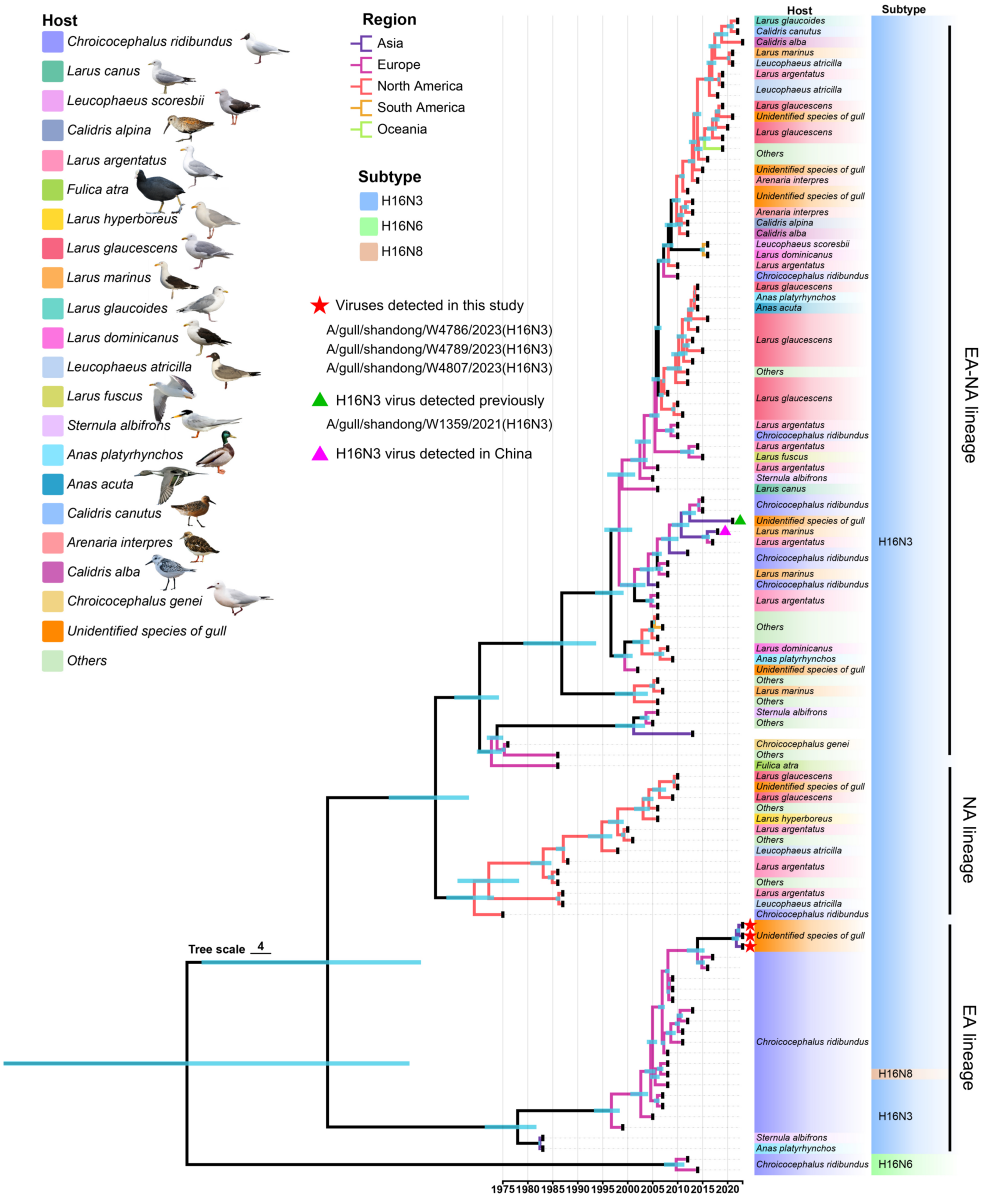
Phylogenetic tree of the global H16 viruses, including three viruses detected in this study, constructed on the basis of HA genes (*n* = 109). The full-length HA sequences were retrieved from GenBank and GISAID. The three viruses used in this study and one virus detected previously are marked in the tree.

To fully understand the evolutionary landscape of H16 viruses, a time-scaled phylogenetic tree of HA genes were constructed by BEAST. All the available HA containing the ORF sequences were initially used to construct a primary time-scaled phylogenetic tree (*n* = 371). Then, the representative strains (*n* = 109) were selected according to their isolation time and region, host species, and the similar identity to present the phylogenetic landscape of HA. Overall, the HA genes of the global H16 viruses have evolved into Eurasian (EA), North American (NA), and Eurasian-North American (EA-NA) lineages, as shown in the phylogenetic tree (Figure 3). Unlike the EA lineage and NA lineage, which are mainly detected in Eurasia and North America, respectively, the EA-NA lineage is detected in various areas of the world (Figure 3). We summarized the strain number of the three lineages and found that the viruses of EA-NA lineage accounts for 67.12% of all the global H16 viruses, while the EA lineage and NA lineage account for 25.88 and 6.47%, respectively (Supplementary Table S3 and Supplementary Figure S3). Importantly, the host species of EA-NA lineage viruses are more variable, including different species of gulls, *Anseriformes* and a common coot (*Fulica atra*), suggesting that the EA-NA lineage is the dominant lineage of the circulating H16 viruses (Figure 3). The HA genes of the three H16N3 viruses detected in this study and the H16N3 and H16N8 viruses detected in Europe clustered in the EA lineage. Interestingly, the H16N3 virus previously detected from gulls in the same wetland in 2021 shared low genetic similarity with the three H16N3/2023 isolates and clustered into the EA-NA lineage (Figure 3).

**Figure 4:**
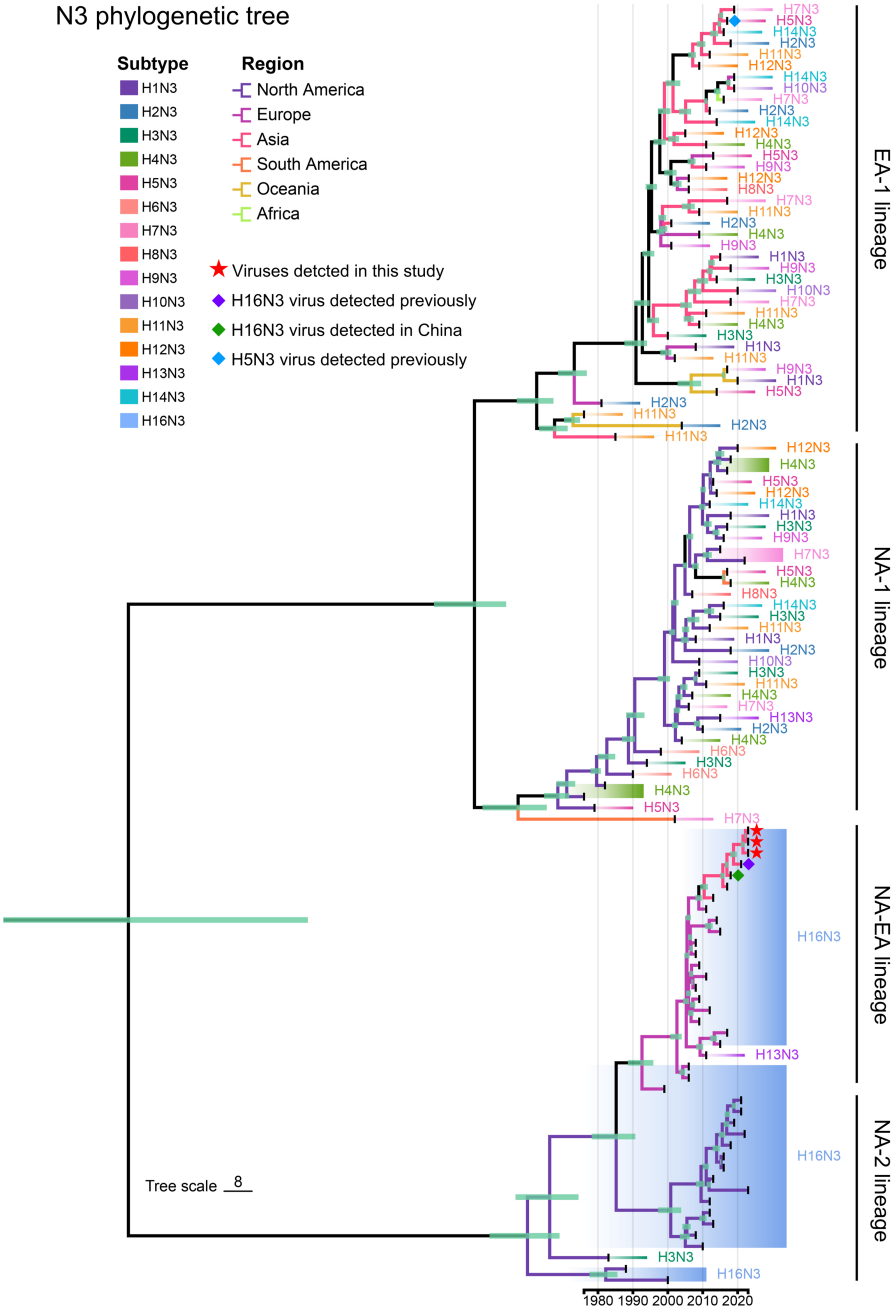
Phylogenetic diversity of NA (N3) genes. A maximum clade credibility (MCC) tree of NA genes (*n* = 114) was constructed from the NA nucleotide sequences of HxN3 (H1–H14) viruses (*n* = 75) and H16N3 viruses (*n* = 39). The three H16N3 viruses detected in this study and the H16N3 and H5N3 viruses previously detected in gulls residing in the YRD wetland are marked with symbols.

We then retrieved the NA nucleotide sequences (*n* = 114) of the HxN3 (H1-H14) viruses and H16N3 viruses to construct an MCC tree of NA genes to reveal the evolutionary trend of viruses of the N3 subtype. The H16N3 viruses clearly formed two distinct lineages (NA-2 and NA-EA lineages), which are significantly different from the HxN3 (H1–H14) viruses (EA-1 and NA-1 lineages). The NA genes of the three H16N3 isolates and the viruses detected in Asia shared high genetic identity and clustered in the NA-EA lineage (Figure 4).

**Figure 5:**
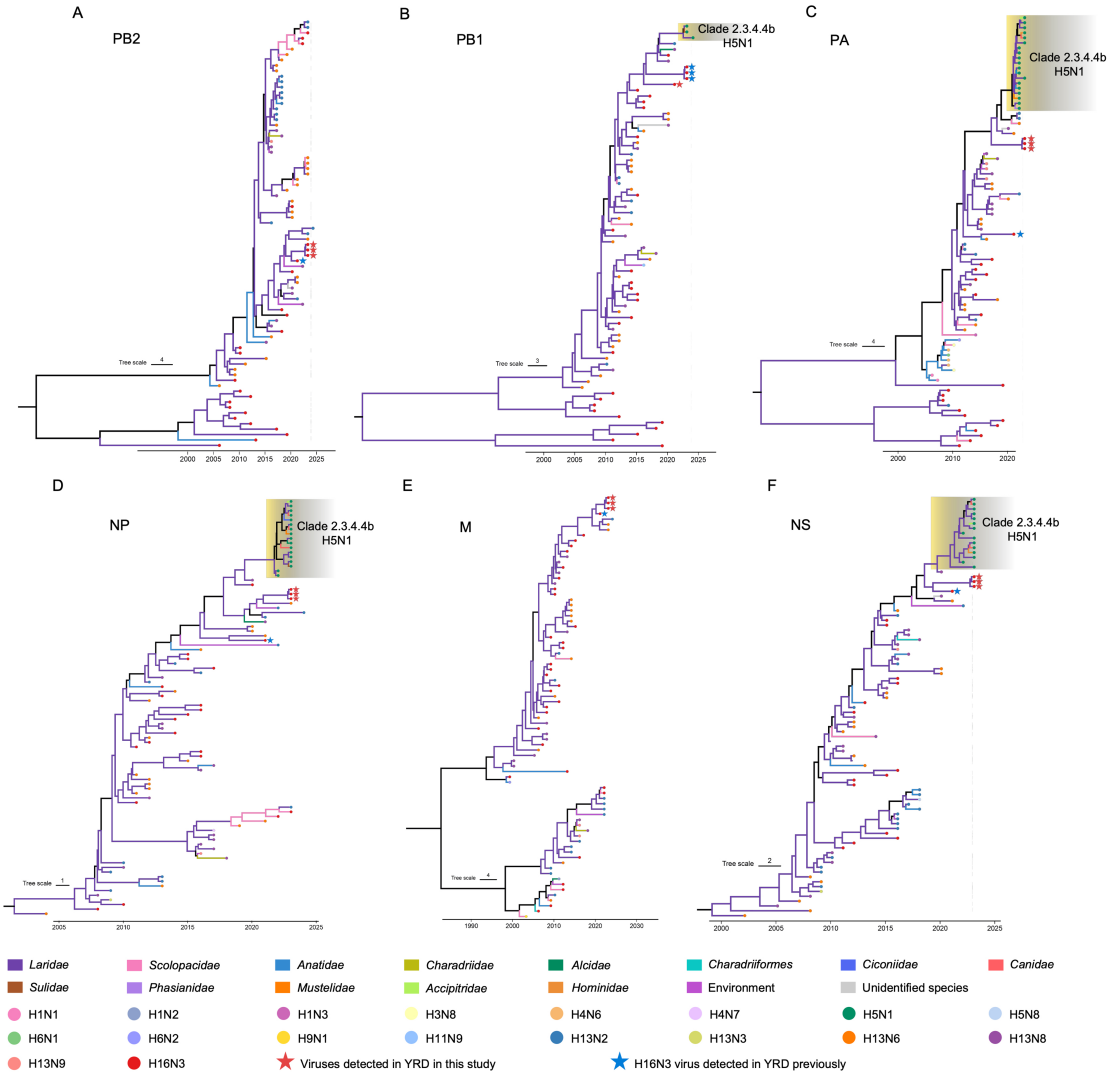
Phylogenetic trees of the internal genes of the H16 viruses. **(A)** PB2 (*n* = 79), **(B)** PB1 (*n* = 73), **(C)** PA (*n* = 86), **(D)** NP (*n* = 90), **(E)** M (*n* = 79), **(F)** NS (*n* = 87). The viruses detected in this study were specifically noted in the phylogenetic trees. The detailed trees containing the virus names are provided in Supplementary Figure S2.

**Figure 6:**
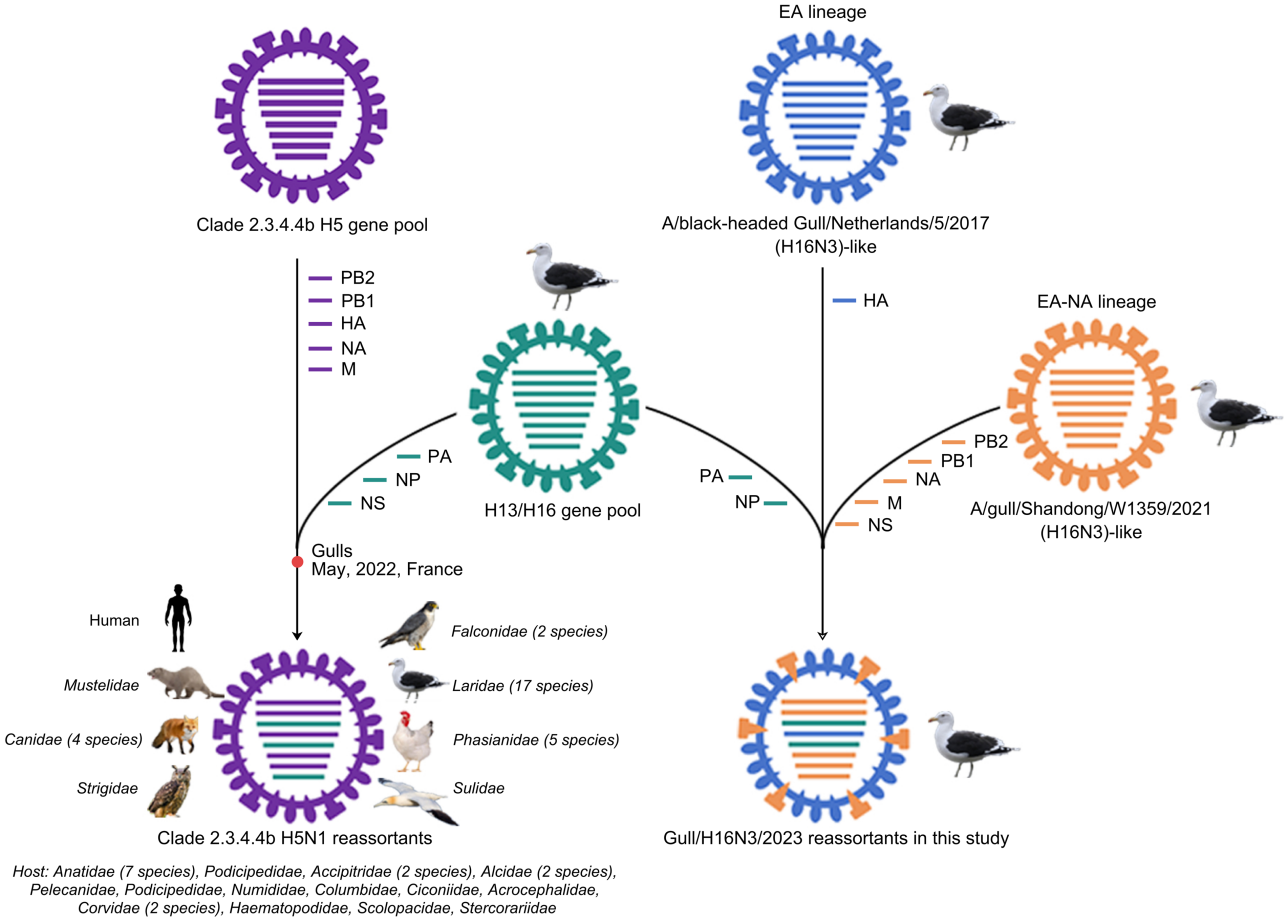
Diagram of gene reassortment events of H16N3 viruses. The gene segments are represented by horizontal bars with different colors (PB2, PB1, PA, HA, NP, NA, M, NS). The possible emerged time point of the H5N1 reassortant in gulls was indicated. The listed families indicate the reassorted H5N1 viruses have been detected at least 55 kinds of species, including 17 family of birds and two family of mammals.

To reveal the evolutionary trends of the internal genes, the PB2, PB1, PA, NP, M, and NS nucleotide sequences of the H16N3 viruses and related viruses were retrieved to construct phylogenetic trees (Supplementary Figure S4). The six internal genes presented high genetic similarity at the nucleotide level (99.9–100, 100, 100%, 99.9–100%, 99.9–100%, and 99.9–100%, respectively), suggesting that the three H16N3 isolates originated from a potential common ancestor. Generally, the referred strains in the phylogenetic trees of the six internal genes dominantly originated from the H13 and H16 viruses, implying that both subtypes share an internal gene cassette (Figure 5 and Supplementary Figure S4). However, H13/H16-like genes, PA, NP and NS, have been introduced into the current circulating clade 2.3.4.4b H5N1 viruses, which have been detected in masses of wild and domestic birds, resulting in occasional mammal and human infections. The reassortment event between the antecedent clade 2.3.4.4b H5N1 virus and the H13/H16-like genes was most likely occurred in gulls, because the reassorted H5N1 virus was first emerged in gulls of France in May 2022 according to the available genome sequences in GISAID (Figure 5 and Supplementary Figures S4, S5). Moreover, the PB2, PB1 and M genes of the three H16N3 isolates clustered together with those of the H13 and H16 viruses, which were detected mostly from different species of gulls in Asia and Europe (Figures 5, 6). Interestingly, although the three H16N3 isolates and the H16N3/2021 virus were isolated at the same location, several genes, especially the PA gene, had significantly different sequences (Figure 5). These phylogenetic analyses indicate that the circulating H16N3 viruses have not only undergone frequent gene reassortment but also contributed to the emergence of highly pathogenic H5N1 viruses as gene donors.

## 4 Discussion

In this study, we isolated three novel H16N3 viruses from migratory gulls in eastern China in 2023, which were different from previous H16 isolates. The host divergence and genetic characteristics of the currently circulated H16N3 viruses may contribute to our better understanding of H16 viruses.

Migratory birds are considered major sources of novel AIVs that have emerged in recent years (Fei and Huang, 2024). The global migration of wild birds further drives the spillover and transmission of viruses in both wild and domestic birds (Hill et al., 2022; Caserta et al., 2024). Emerging novel AIVs, such as the highly pathogenic H5N1 virus and the lowly pathogenic H3N8 and H7N4 viruses, which originate wholly or partially from wild bird viruses, have been frequently found to spillover to humans (Peacock et al., 2024; Cui et al., 2023; Chai et al., 2022). However, there is still a large knowledge gap concerning the ecology, epidemiology and evolution of AIVs, which are found primarily in wild birds.

Unlike the common subtypes commonly found in domestic ducks and chickens, H16 viruses are found primarily in gulls, resulting in limited information concerning their prevalence and evolution (Verhagen et al., 2020; Poen et al., 2017; Verhagen et al., 2015). The detailed analysis of the host divergence of the seven rare subtypes suggests that the waterfowl of *Anatidae* are the primary host reservoirs of the H8, H11, H12, H14, and H15 subtypes, whereas the H13 and H16 subtypes share gulls as reservoirs. Our previous studies also revealed that rare subtypes, such as H8, H11 and H12, were predominantly pooled in *Anatidae*, whereas H16N3 viruses mostly originated from gulls (Wang et al., 2022; Wang et al., 2024; Zhang et al., 2024; Shen et al., 2024). A bioinformatics study reported signatures of the internal proteins, such as lack of one nuclear localization signal in NS1 protein, are possibly related to host restriction of H13 and H16 AIVs (Tønnessen et al., 2013). As common hosts for H13 and H16 subtypes, different species of gulls may facilitate frequent gene flow or segment exchanges between these viruses because extensive gene segment (PB2, PB1, PA, NP, M, and NS) reassortment was observed via genetic analysis.

The AIVs of each subtype are generally classified into the EA lineage or the NA lineage according to their phylogenetic differences and isolation regions. However, viruses of the EA or NA lineage can occasionally be detected beyond their prevalent regions. In a previous study, we reported that the NA lineage of H10 viruses was introduced into Asia by migratory birds and established a unique sublineage in Bangladesh, South Korea and China in 2019 and 2020 (Wang et al., 2022). The coexistence of the EA, NA and EA-NA lineages of H16 viral HA genes revealed significant divergence of the global H16 viruses in different species of gulls. The dominance of the EA-NA lineage indicated that the H16 viruses of the EA and NA lineages have undergone frequent gene exchanges between Eurasia and North America in the past two decades. As the primary natural reservoir of H16 viruses, gulls travel much longer distances than *Anseriformes* do (Hill et al., 2022; Arnal et al., 2015), which may drive the global dissemination of EA-NA lineage viruses. The HA genes of the three H16N3/2023 viruses identified in this study were significantly different from the H16N3/2021 virus and clustered in the EA lineage, although they were isolated from the same wetland in eastern China. The high similarity of the NA, PB2, PB1, M and NS genes of the H16N3 viruses in the YRD wetland implied that the novel H16N3/2023 viruses partially originated from the H16N3/2021 virus via segment reassortment with EA lineage viruses. The replication and pathogenicity of AIVs in mammals is determined by multiple factors, including key amino acid substitutions at a specific gene segment or the synergistic action of multiple gene segments. Although several amino acid substitutions that contribute to the increased replication and pathogenicity of H5N1 and H7N9 viruses in mammals have been observed in all or almost all H16 viruses, the previously reported H16 virus exhibited poor adaptation in mammalian models (Wang et al., 2022). More detailed studies are needed to evaluate the mammalian adaptation of the H16 viruses and further uncover the molecular basis.

The currently circulating highly pathogenic clade 2.3.4.4b H5N1 viruses have been a public concern because of their widespread dissemination in both birds and mammals. Since the emergence of H5N8 viruses in Europe in 2019, extensive genetic reassortment events have facilitated the emergence and evolution of clade 2.3.4.4b H5N1 viruses (Cui et al., 2022; Xing et al., 2024; Tian et al., 2021; Cui et al., 2022). Moreover, massive infections and mortality of different species of gulls worldwide have confirmed the incursion of H5N1 viruses into the gulls (Taylor et al., 2023; Kareinen et al., 2024; Caliendo et al., 2024; Caliendo et al., 2022). Here, we identified the H13/H16-like PA, NP, and NS genes have been introduced into the highly pathogenic clade 2.3.4.4b H5N1 viruses since May 2022. The gulls may play a key role as “mixture” for the circulated H5N1 and H13 or H16 viruses, because the emerged H5N1 reassortant was first detected in gulls of France according to the public H5N1 sequences and the phylogenetic analysis in this study. To date, these H5N1 reassortants have been detected in mink, fox, dog and even human samples, implying their increased globally public risk. Additionally, further studies were essential to identify whether these H13/H16-like genes contributed to replication or cross-species transmission of the emerged H5N1 reassortants.

In conclusion, our detailed genetic analysis identified three novel gull H16N3 viruses to reveal the evolutionary dynamics of H16 viruses. The genetic diversity and frequent gene reassortment of H16 viruses further emphasize the need for active surveillance in migratory birds to monitor the global dissemination of AIVs.

## Data Availability

The whole genome sequences of the tree H16N3 viruses have been deposited in GISAID (https://gisaid.org) and the accession number is: EPI3605848-EPI3605855, EPI3605910-EPI3605915, EPI3606284-EPI3606293.

## Footnotes

^1^ https://www.chiplot.online/tvbot.html
