# Supplementary material for: Novel H16N3 avian influenza viruses isolated from migratory gulls in China in 2023
Source: Front Microbiol. 2025 Jan 24;15:1543338. doi: 10.3389/fmicb.2024.1543338 (PMC11802517; doi:10.3389/fmicb.2024.1543338)

Supporting Information

Table S1. Information on the three H16N3 viruses used in this study.

Table S2. List of species of birds and mammals mentioned in this study. Bird names taxonomy follows the Clements Checklist.

Table S3. The global H16 viruses of EA lineage, NA lineage, and EA-NA lineage.

Figure S1. Figure S1. Location of the sampling site and the gulls at YRD wetland.

Figure S2. Number of each HA subtype (H1-H16) in GISAID. The number of each subtype retrieved from GISAID was raw data and not checked to remove the duplicate sequences.

Figure S3. Trend analysis of the EA, NA, EA-NA lineages of H16 viruses since 1975.

Figure S4. Detailed phylogenetic trees of the internal genes (PB2, PB1, PA, NP, M, and NS) of the H16N3 viruses.

Figure S5. Bayesian timed resolved tree of the PA gene of the circulated H5N1 viruses between January 2022 to May 2022.

Table S1. Information on the three H16N3 viruses used in this study.

NO.	Full name	Abbreviation	Subtype	Location	Collection Date	Host	Specimen source	Sample numbers
1	A/gull/Shandong/W4786/2023	GL/W4786/23	H16N3	Yellow River Delta wetland, Shandong, China	24 Oct 2023	Gull	Fecal droppings	1342
2	A/gull/Shandong/W4789/2023	GL/W4789/23	H16N3					
3	A/gull/Shandong/W4807/2023	GL/W4807/23	H16N3					

Table S2. List of species of birds and mammals mentioned in this study. Bird names taxonomy follows the Clements Checklist.

Accipitriformes	*Accipitridae*	*Accipiter nisus*	Eurasian sparrowhawk, Sparrowhawk
		Buteo buteo	Buteo buteo, Common buzzard
Anseriformes	*Anatidae*	*Aix galericulata*	Mandarin duck
		Aix sponsa	Wood duck
		Anas	Spotbill duck
		Anas acuta	Northern pintail
		Anas clypeata	Anas clypeata
		Anas crecca	Common teal, Green-winged Teal, American green winged teal
		Anas erythrorhyncha	Red billed teal
		Anas flavirostris	Yellow billed teal
		Anas georgica	Yellow billed pintail
		Anas gracilis	Grey teal
		Anas plathyrhynchos	Anas plathyrhynchos
		Anas platyrhynchos	Mallard, Mallard duck
		Anas platyrhynchos (Domestic type)	Duck, Domestic duck, call duck, peking duck
		Anas platyrhynchos x rubripes	Mallard black duck hybrid, Mallard x black duck
		Anas poecilorhyncha	Spot billed duck
		Anas rubripes	American black duck, Black duck, common black duck
		Anas superciliosa	Pacific black duck
		Anser albifrons	White fronted goose
		Anser anser	Anser anser
		Anser anser (Domestic type)	Domestic goose, Goose
		Anser brachyrhynchus	Anser brachyrhynchus
		Anser caerulescens	Greater snow goose, Lesser snow goose
		Anser canagicus	Emperor goose
		Anser fabalis	Bean goose
		Anser indicus	Bar headed goose
		Anser rossii	Rosss goose
		Aythya affinis	Lesser scaup
		Aythya americana	Redhead duck
		Aythya collaris	Ring necked duck
		Aythya ferina	Pochard
		Aythya marila	Greater scaup
		Aythya valisineria	Canvasback cuck

		Branta bernicla	Brant
		Branta canadensis	Canada goose
		Bucephala albeola	Bufflehead
		Bucephala clangula	Common goldeneye
		Cairina moschata	Muscovy duck
		Chenonetta jubata	Australian wood duck
		Clangula hyemalis	Long tailed duck
		Cygnus columbianus bewickii	Bewick swan
		Cygnus columbianus columbianus	Whistling swan
		Cygnus cygnus	Swan, Whooper swan
		Cygnus olor	Mute swan
		Dendrocygna javanica	Lesser whistling duck
		Heteronetta atricapilla	Black headed duck
		Lophodytes cucullatus	Hooded merganser
		Mareca americana	American wigeon
		Mareca falcata	Anas falcata
		Mareca penelope	Eurasian wigeon
		Mareca strepera	Gadwall
		Melanitta deglandi	White winged scoter
		Melanitta perspicillata	Surf scoter
		Polysticta stelleri	Stellers eider
		Sibirionetta formosa	Baikal teal
		Somateria mollissima	Common eider
		Spatula clypeata	Northern shoveler
		Spatula cyanoptera	Cinnamon teal
		Spatula discors	Blue winged teal
		Spatula platalea	Red shoveler
		Spatula querquedula	Garganey
		Tadorna ferruginea	Ruddy shelduck
		Tadorna tadorna	Common shelduck
		Tadorna tadornoides	Australian shelduck
		Unidentified species	Anser, chilean teal, pintail, pintail duck, shelduck, shoveler, teal, waterfowl, wild duck, wild goose, wild waterfowl, widgeon
Charadriiformes	*Alcidae*	*Alca torda*	Raxorbill
		Uria aalge	Common guillemot, Common murre,

			guillemot
		Uria lomvia	Thick billed murre
	Charadriidae	*Vanellus chilensis*	Southern lapwing
	Chionidae	*Chionis albus*	Snowy sheathbill
	Haematopodidae	*Haematopus palliatus*	American oystercatcher
		Haematopus ater	Blackish oystercatcher
		Haematopus ostralegus	Oystercatcher, Eurasian oystercatcher
	Laridae	*Chroicocephalus genei*	Slender billed gull
		Chroicocephalus maculipennis	Brown hooded gull
		Chroicocephalus novaehollandiae	Silver gull
		Chroicocephalus ridibundus	Black headead gull, Larus ridibundus
		Gelochelidon nilotica	Gull billed tern
		Hydrocoloeus minutus	Little gull, Larus minutus
		Ichthyaetus audouinii	Audouins gull
		Ichthyaetus ichthyaetus	Larus ichthyaetus
		Ichthyaetus melanocephalus	Mediterranean gull
		Larus argentatus	European herring gull, Herring gull
		Larus armenicus	Armenian gull
		Larus cachinnans	Caspian gull
		Larus californicus	California gull
		Larus canus	Mew gull, Common gull
		Larus crassirostris	Black tailed gull
		Larus delawarensis	Ring billed gull
		Larus dominicanus	Kelp gull
		Larus fuscus	Lesser black backed gull
		Larus glaucescens	Glaucous winged gull
		Larus glaucoides	Iceland gull
		Larus hyperboreus	Glaucous gull
		Larus marinus	Great black backed gull, Great black headed gull
		Larus michahellis	Yellow legged gull
		Larus mongolicus	Mongolian gull
		Larus schistisagus	Slaty backed gull
		Larus smithsonianus	American herring gull

		Larus smithsonianus x hyperboreus	Glaucous gull herring gull hybrid
		Leucophaeus atricilla	Laughing gull
		Leucophaeus pipixcan	Franklins gull
		Leucophaeus scoresbii	Dolphin gull
		Onychoprion fuscatus	Sooty tern
		Rissa tridactyla	Black legged kittiwake, Kittiwake
		Rynchops niger	Black skimmer
		Sterna hirundo	Common tern
		Sterna paradisaea	Arctic tern
		Sternula albifrons	Little tern
		Thalasseus elegans	Elegant tern
		Thalasseus sandvicensis	Sandwich tern
		Unidentified species	Gull, seagull, tern
	Recurvirostridae	*Himantopus mexicanus melanurus*	Black necked stilt, White backed stilt
	Scolopacidae	*Arenaria interpres*	Ruddy turnstone
		Calidris acuminata	Sharp tailed sandpiper
		Calidris alba	Sanderling
		Calidris alpina	Dunlin
		Calidris canutus	Red knot
		Calidris fuscicollis	White rumped sandpiper
		Calidris minutilla	Least sandpiper
		Calidris pusilla	Semipalmated sandpiper
		Calidris ruficollis	Red necked stint
		Gallinago gallinago	Common snipe
		Limnodromus griseus	Short billed dowitcher
		Numenius arquata	Curlew, Eurasian curlew
		Numenius phaeopus	Whimbrel
		Tringa glareola	Wood sandpiper
		Unidentified species	Knot, sandpiper
	Stercorariidae	*Stercorarius parasiticus*	Parasitic jaeger
	Unidentified species	*Unidentified species*	Shorebird
Ciconiiformes	*Ciconiidae*	*Ciconia boyciana*	Oriental white stork
		Ciconia ciconia	White stork
Columbiformes	*Columbidae*	*Unidentified species*	Pigeon

Falconiformes	*Falconidae*	*Falco tinnunculus*	Common kestrel
		Falco peregrinus	Peregrine falcon
Galliformes	*Phasianidae*	*Gallus gallus*	Chicken, Korean native chicken
	Numididae	*Unidentified species*	Guineafowl
		Numida meleagris	Helmeted guineafowl
	Phasianidae	*Unidentified species*	Partridge
		Pavo cristatus	Pavo cristatus
		Phasianus colchicus	Pheasant, Ring-necked pheasant
		Meleagris gallopavo	Turkey
Gruiformes	*Gruidae*	*Unidentified species*	Crane
	Rallidae	*Fulica atra*	Eurasian coot
		Gallicrex cinerea	Watercock
Passeriformes	*Corvidae*	*Coloeus monedula*	Corvus monedula
		Pica pica	Eurasian magpie, Common magpie
	Acrocephalidae	*Acrocephalus scirpaceus*	Reed warbler, Common reed warbler
Pelecaniformes	*Pelecanidae*	*Pelecanus erythrorhynchos*	American white pelican
	Ardeidae	*Egretta eulophotes*	Egret
	Pelecanidae	*Unidentified species*	Pelican
Phoenicopteriformes	*Phoenicopteridae*	*Phoeniconaias minor*	Flamingo, Lesser flamingo
Podicipediformes	*Podicipedidae*	*Podiceps cristatus*	Great crested grebe
Primates	*Hominidae*	*Homo sapiens*	Human
Procellariiformes	*Procellariidae*	*Unidentified species*	Shearwater
		Ardenna pacifica	Wedge tailed shearwater
Rheiformes	*Rheidae*	*Rhea americana*	Greater rhea
Sphenisciformes	*Spheniscidae*	*Pygoscelis adeliae*	Adelie penguin
		Pygoscelis antarcticus	Chinstrap penguin
		Pygoscelis papua	Gentoo penguin
Strigiformes	*Strigidae*	*Bubo bubo*	Eurasian eagle owl, Eagle owl
Struthioniformes	*Struthionidae*	*Struthio camelus*	Ostrich
Suliformes	*Sulidae*	*Morus bassanus*	Northern gannet
Artiodactyla	*Suidae*	*Sus scrofa*	Swine
Carnivora	*Canidae*	*Vulpes lagopus*	Arctic fox
		Unidentified species	Blue fox
		Unidentified species	Fox
	Mustelidae	*Unidentified species*	Mink
	Canidae	*Nyctereutes procyonoides*	Raccoon dog
		Vulpes vulpes	Red fox

		Vulpes fulva	Silver fox
	Mustelidae	*Unidentified species*	White mink
Cetacea	*Delphinidae*	*Unidentified species*	Pilot whale
	Cetacea	*Unidentified species*	Whale
Unidentified species	*Unidentified species*	*Unidentified species*	Aquatic bird, avian, bird, daw, ibis, madagascar, she, wild bird, wild migratory bird, wild waterbird
Environment	*Environment*	*Environment*	Environment, Feces

Table S3. The global H16 viruses of EA lineage, NA lineage, and EA-NA lineage.

	Percentage	Region	Host	Subtype
EA-NA lineage	67.12%	North America (65.86%), Europe (26.10%), South America (4.42%), Asia (2.81%), Oceania (0.80%)	Laridae (87.15%), Environment (4.02%), Scolopacidae (3.21%), Anatidae (2.01%), Unidentified species (2.01%), Charadriiformes (1.20%), Rallidae (0.40%)	H16N3 (97.59%), H16 (2.41%)
NA lineage	6.47%	North America (100%)	Laridae (62.50%), Scolopacidae (4.17%), Anatidae (4.17%), Environment (25.00%), Charadriiformes (4.17%)	H16N3 (83.3%), H16 (16.7%)
EA lineage	25.88%	Asia (96.88%), Europe (3.13%)	Laridae (98.96%), Anatidae (1.04%)	H16N8 (97.92%), H16 (1.04%), H16N3 (1.04%)

Figure S1

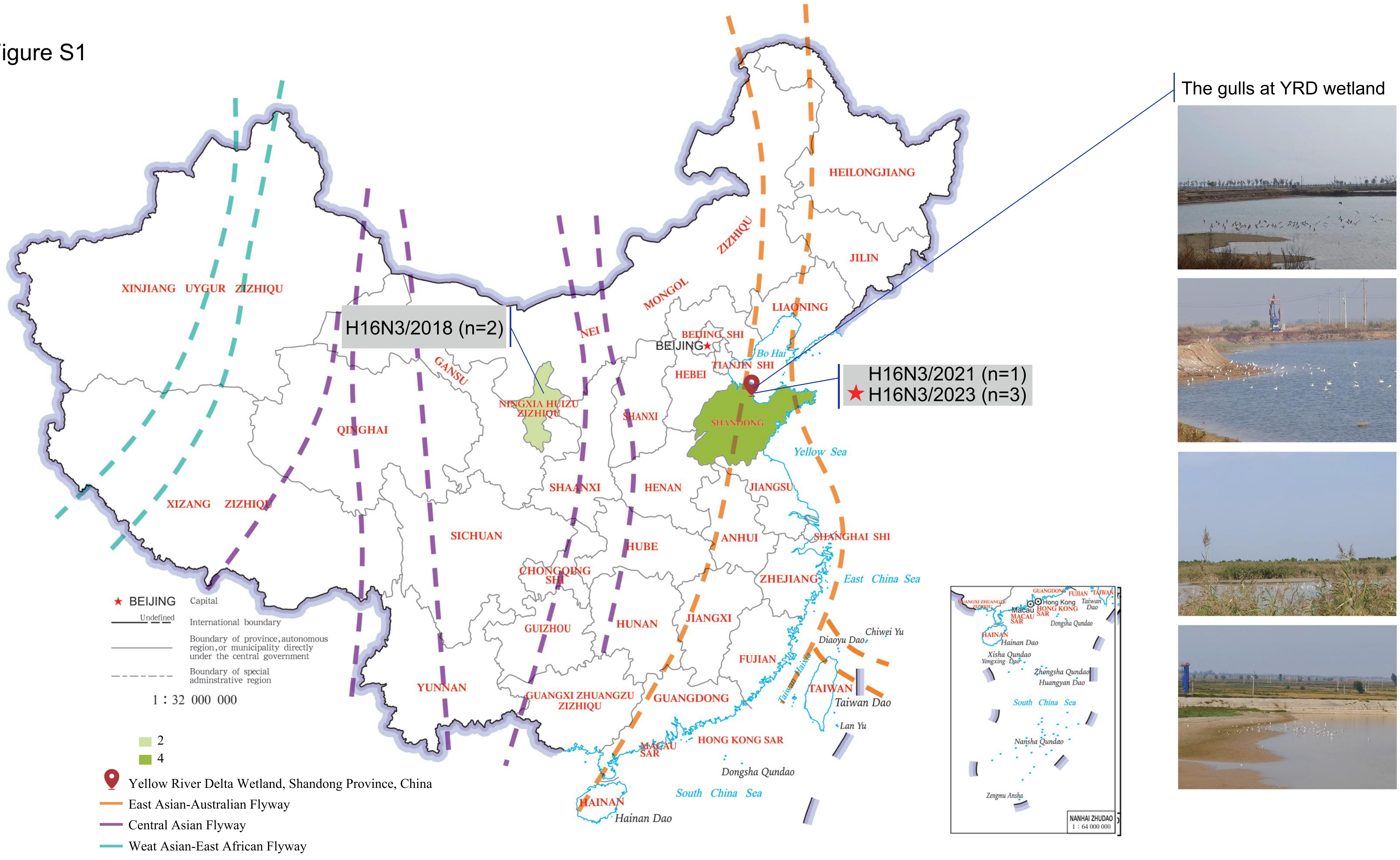

The gulls at YRD wetland
H16N3/2018 (n=2)
H16N3/2021 (n=1)
★ H16N3/2023 (n=3)
XINJIANG UYGUR ZIZHIQU
XIZANG ZIZHIQU
QINGHAI
GANSU
NINGXIA HUIZU ZIZHIQU
NEI MONGOL ZIZHIQU
HEILONGJIANG
JILIN
LIAONING
BEIJING SHI
BEIJING★
TIANJIN SHI
HEBEI
SHANXI
SHANDONG
SHAANXI
HENAN
JIANGSU
ANHUI
SHANGHAI SHI
SICHUAN
HUBE
CHONGQING SHI
ZHEJIANG
GUIZHOU
HUNAN
JIANGXI
FUJIAN
YUNNAN
GUANGXI ZHUANGZU ZIZHIQU
GUANGDONG
TAIWAN
Taiwan Dao
Lan Yu
Chiwei Yu
Diaoyu Dao
Taiwan Haixia
HONG KONG SAR
MACAU SAR
HAINAN
Hainan Dao
Dongsha Qundao
South China Sea
East China Sea
Yellow Sea
Bo Hai
★ BEIJING Capital
International boundary
Undefined
Boundary of province, autonomous region, or municipality directly under the central government
Boundary of special adminstrative region
1 : 32 000 000
2
4
Yellow River Delta Wetland, Shandong Province, China
East Asian-Australian Flyway
Central Asian Flyway
Weat Asian-East African Flyway
Hong Kong
Macau
Xisha Qundao
Yongxing Dao
Zhongsha Qundao
Huangyan Dao
Nansha Qundao
Zengmu Ansha
NANHAI ZHUDAO
1 : 64 000 000

Figure S2

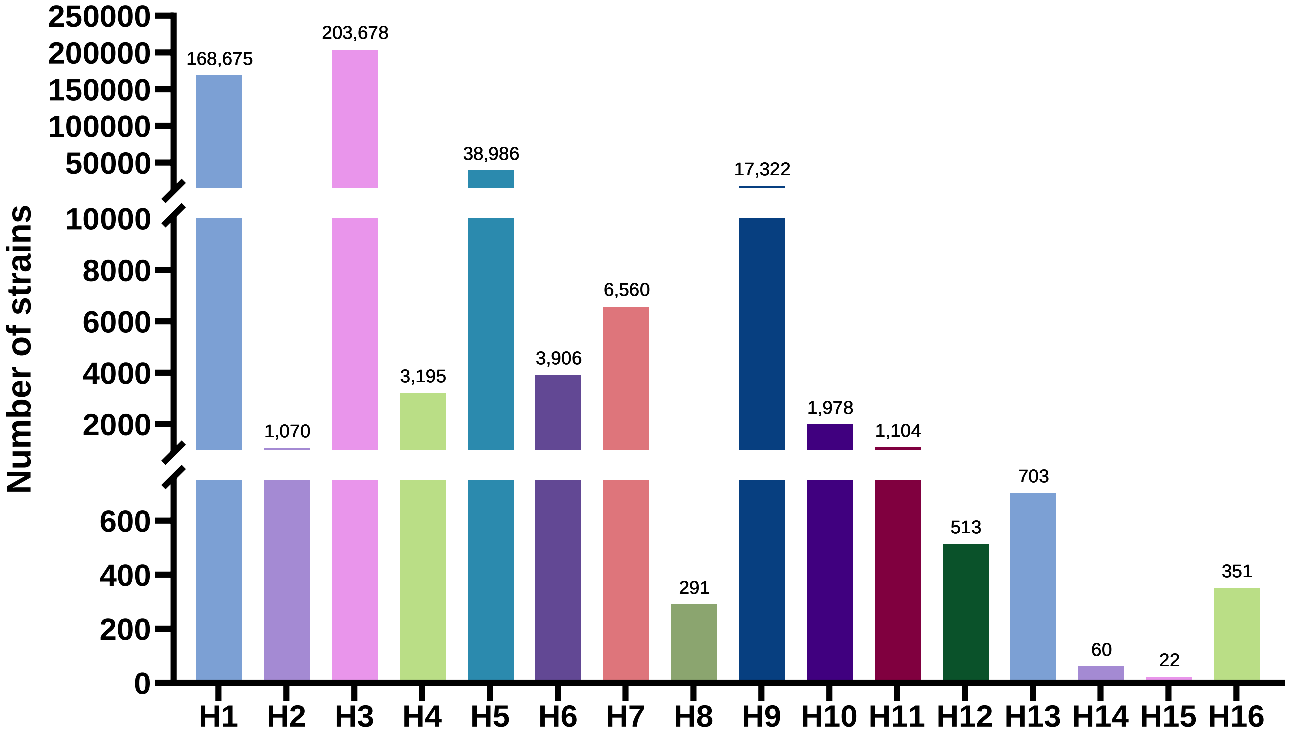
Number of strains
250000
200000
150000
100000
50000
10000
8000
6000
4000
2000
600
400
200
0
168,675
1,070
203,678
3,195
38,986
3,906
6,560
291
17,322
1,978
1,104
513
703
60
22
351
H1
H2
H3
H4
H5
H6
H7
H8
H9
H10
H11
H12
H13
H14
H15
H16

Figure S3

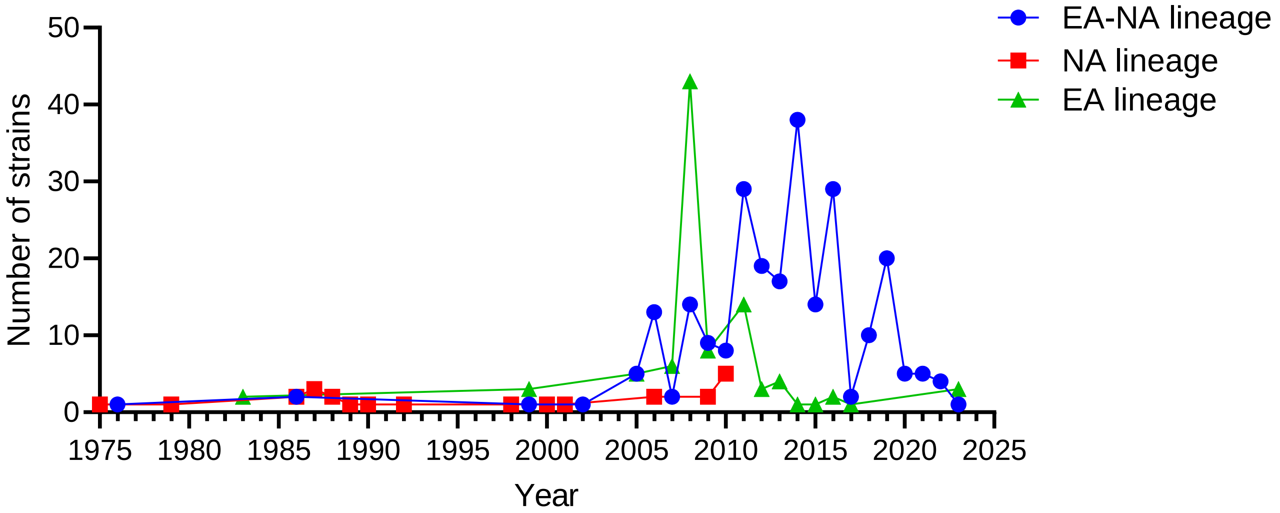

EA-NA lineage
NA lineage
EA lineage
Number of strains
0
10
20
30
40
50
1975
1980
1985
1990
1995
2000
2005
2010
2015
2020
2025
Year

Figure S4

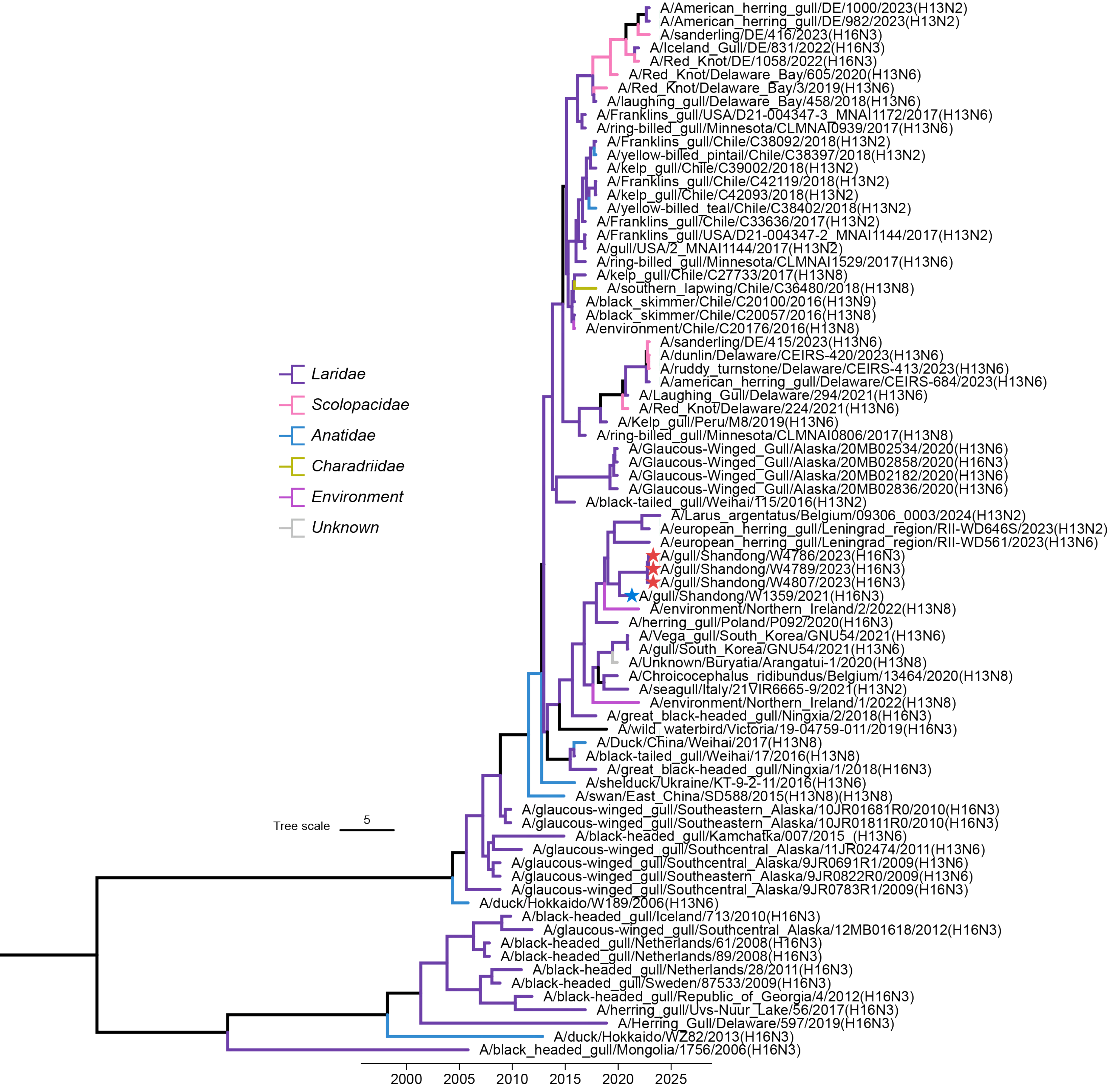

A/American_herring_gull/DE/1000/2023(H13N2)
A/American_herring_gull/DE/982/2023(H13N2)
A/sanderling/DE/416/2023(H16N3)
A/Iceland_Gull/DE/831/2022(H16N3)
A/Red_Knot/DE/1058/2022(H16N3)
A/Red_Knot/Delaware_Bay/605/2020(H13N6)
A/Red_Knot/Delaware_Bay/3/2019(H13N6)
A/laughing_gull/Delaware_Bay/458/2018(H13N6)
A/Franklins_gull/USA/D21-004347-3_MNAI1172/2017(H13N6)
A/ring-billed_gull/Minnesota/CLMNAI0939/2017(H13N6)
A/Franklins_gull/Chile/C38092/2018(H13N2)
A/yellow-billed_pintail/Chile/C38397/2018(H13N2)
A/kelp_gull/Chile/C39002/2018(H13N2)
A/Franklins_gull/Chile/C42119/2018(H13N2)
A/kelp_gull/Chile/C42093/2018(H13N2)
A/yellow-billed_teal/Chile/C38402/2018(H13N2)
A/Franklins_gull/Chile/C33636/2017(H13N2)
A/Franklins_gull/USA/D21-004347-2_MNAI1144/2017(H13N2)
A/gull/USA/2_MNAI1144/2017(H13N2)
A/ring-billed_gull/Minnesota/CLMNAI1529/2017(H13N6)
A/kelp_gull/Chile/C27733/2017(H13N8)
A/southern_lapwing/Chile/C36480/2018(H13N8)
A/black_skimmer/Chile/C20100/2016(H13N9)
A/black_skimmer/Chile/C20057/2016(H13N8)
A/environment/Chile/C20176/2016(H13N8)
A/sanderling/DE/415/2023(H13N6)
A/dunlin/Delaware/CEIRS-420/2023(H13N6)
A/ruddy_turnstone/Delaware/CEIRS-413/2023(H13N6)
A/american_herring_gull/Delaware/CEIRS-684/2023(H13N6)
A/Laughing_Gull/Delaware/294/2021(H13N6)
A/Red_Knot/Delaware/224/2021(H13N6)
A/Kelp_gull/Peru/M8/2019(H13N6)
A/ring-billed_gull/Minnesota/CLMNAI0806/2017(H13N8)
A/Glaucous-Winged_Gull/Alaska/20MB02534/2020(H13N6)
A/Glaucous-Winged_Gull/Alaska/20MB02858/2020(H16N3)
A/Glaucous-Winged_Gull/Alaska/20MB02182/2020(H13N6)
A/Glaucous-Winged_Gull/Alaska/20MB02836/2020(H13N6)
A/black-tailed_gull/Weihai/115/2016(H13N2)
A/Larus_argentatus/Belgium/09306_0003/2024(H13N2)
A/european_herring_gull/Leningrad_region/RII-WD646S/2023(H13N2)
A/european_herring_gull/Leningrad_region/RII-WD561/2023(H13N6)
A/gull/Shandong/W4786/2023(H16N3)
A/gull/Shandong/W4789/2023(H16N3)
A/gull/Shandong/W4807/2023(H16N3)
A/gull/Shandong/W1359/2021(H16N3)
A/environment/Northern_Ireland/2/2022(H13N8)
A/herring_gull/Poland/P092/2020(H16N3)
A/Vega_gull/South_Korea/GNU54/2021(H13N6)
A/gull/South_Korea/GNU54/2021(H13N6)
A/Unknown/Buryatia/Arangatui-1/2020(H13N8)
A/Chroicocephalus_ridibundus/Belgium/13464/2020(H13N8)
A/seagull/Italy/21VIR6665-9/2021(H13N2)
A/environment/Northern_Ireland/1/2022(H13N8)
A/great_black-headed_gull/Ningxia/2/2018(H16N3)
A/wild_waterbird/Victoria/19-04759-011/2019(H16N3)
A/Duck/China/Weihai/2017(H13N8)
A/black-tailed_gull/Weihai/17/2016(H13N8)
A/great_black-headed_gull/Ningxia/1/2018(H16N3)
A/shelduck/Ukraine/KT-9-2-11/2016(H13N6)
A/swan/East_China/SD588/2015(H13N8)(H13N8)
A/glaucous-winged_gull/Southeastern_Alaska/10JR01681R0/2010(H16N3)
A/glaucous-winged_gull/Southeastern_Alaska/10JR01811R0/2010(H16N3)
A/black-headed_gull/Kamchatka/007/2015_(H13N6)
A/glaucous-winged_gull/Southcentral_Alaska/11JR02474/2011(H13N6)
A/glaucous-winged_gull/Southcentral_Alaska/9JR0691R1/2009(H13N6)
A/glaucous-winged_gull/Southeastern_Alaska/9JR0822R0/2009(H13N6)
A/glaucous-winged_gull/Southcentral_Alaska/9JR0783R1/2009(H16N3)
A/duck/Hokkaido/W189/2006(H13N6)
A/black-headed_gull/Iceland/713/2010(H16N3)
A/glaucous-winged_gull/Southcentral_Alaska/12MB01618/2012(H16N3)
A/black-headed_gull/Netherlands/61/2008(H16N3)
A/black-headed_gull/Netherlands/89/2008(H16N3)
A/black-headed_gull/Netherlands/28/2011(H16N3)
A/black-headed_gull/Sweden/87533/2009(H16N3)
A/black-headed_gull/Republic_of_Georgia/4/2012(H16N3)
A/herring_gull/Uvs-Nuur_Lake/56/2017(H16N3)
A/Herring_Gull/Delaware/597/2019(H16N3)
A/duck/Hokkaido/WZ82/2013(H16N3)
A/black_headed_gull/Mongolia/1756/2006(H16N3)
Laridae
Scolopacidae
Anatidae
Charadriidae
Environment
Unknown
Tree scale
5
2000
2005
2010
2015
2020
2025

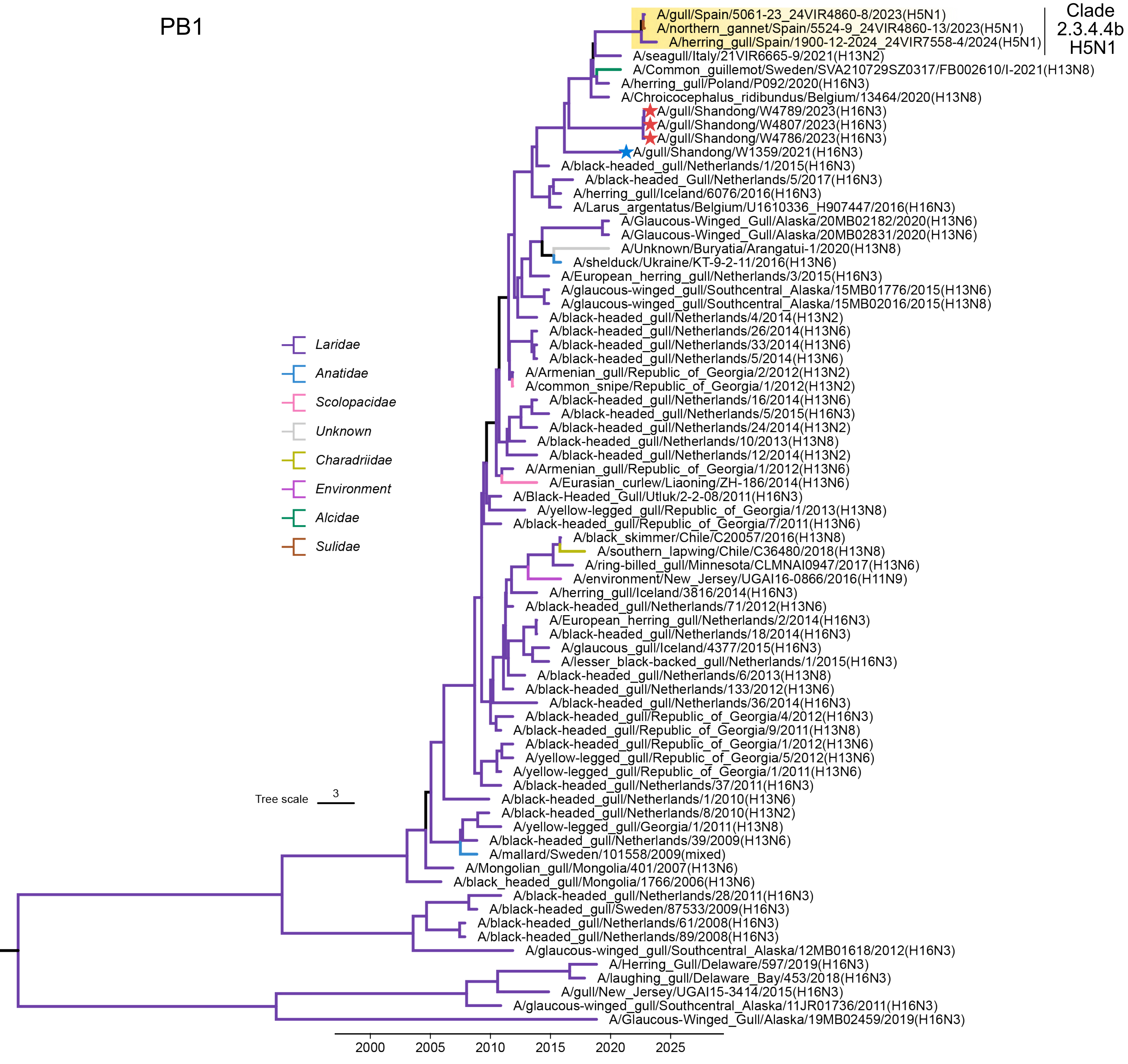
PB1
A/gull/Spain/5061-23_24VIR4860-8/2023(H5N1)
A/northern_gannet/Spain/5524-9_24VIR4860-13/2023(H5N1)
A/herring_gull/Spain/1900-12-2024_24VIR7558-4/2024(H5N1)
Clade 2.3.4.4b H5N1
A/seagull/Italy/21VIR6665-9/2021(H13N2)
A/Common_guillemot/Sweden/SVA210729SZ0317/FB002610/I-2021(H13N8)
A/herring_gull/Poland/P092/2020(H16N3)
A/Chroicocephalus_ridibundus/Belgium/13464/2020(H13N8)
A/gull/Shandong/W4789/2023(H16N3)
A/gull/Shandong/W4807/2023(H16N3)
A/gull/Shandong/W4786/2023(H16N3)
A/gull/Shandong/W1359/2021(H16N3)
A/black-headed_gull/Netherlands/1/2015(H16N3)
A/black-headed_Gull/Netherlands/5/2017(H16N3)
A/herring_gull/Iceland/6076/2016(H16N3)
A/Larus_argentatus/Belgium/U1610336_H907447/2016(H16N3)
A/Glaucous-Winged_Gull/Alaska/20MB02182/2020(H13N6)
A/Glaucous-Winged_Gull/Alaska/20MB02831/2020(H13N6)
A/Unknown/Buryatia/Arangatui-1/2020(H13N8)
A/shelduck/Ukraine/KT-9-2-11/2016(H13N6)
A/European_herring_gull/Netherlands/3/2015(H16N3)
A/glaucous-winged_gull/Southcentral_Alaska/15MB01776/2015(H13N6)
A/glaucous-winged_gull/Southcentral_Alaska/15MB02016/2015(H13N8)
A/black-headed_gull/Netherlands/4/2014(H13N2)
A/black-headed_gull/Netherlands/26/2014(H13N6)
A/black-headed_gull/Netherlands/33/2014(H13N6)
A/black-headed_gull/Netherlands/5/2014(H13N6)
A/Armenian_gull/Republic_of_Georgia/2/2012(H13N2)
A/common_snipe/Republic_of_Georgia/1/2012(H13N2)
A/black-headed_gull/Netherlands/16/2014(H13N6)
A/black-headed_gull/Netherlands/5/2015(H16N3)
A/black-headed_gull/Netherlands/24/2014(H13N2)
A/black-headed_gull/Netherlands/10/2013(H13N8)
A/black-headed_gull/Netherlands/12/2014(H13N2)
A/Armenian_gull/Republic_of_Georgia/1/2012(H13N6)
A/Eurasian_curlew/Liaoning/ZH-186/2014(H13N6)
A/Black-Headed_Gull/Utluk/2-2-08/2011(H16N3)
A/yellow-legged_gull/Republic_of_Georgia/1/2013(H13N8)
A/black-headed_gull/Republic_of_Georgia/7/2011(H13N6)
A/black_skimmer/Chile/C20057/2016(H13N8)
A/southern_lapwing/Chile/C36480/2018(H13N8)
A/ring-billed_gull/Minnesota/CLMNAI0947/2017(H13N6)
A/environment/New_Jersey/UGAI16-0866/2016(H11N9)
A/herring_gull/Iceland/3816/2014(H16N3)
A/black-headed_gull/Netherlands/71/2012(H13N6)
A/European_herring_gull/Netherlands/2/2014(H16N3)
A/black-headed_gull/Netherlands/18/2014(H16N3)
A/glaucous_gull/Iceland/4377/2015(H16N3)
A/lesser_black-backed_gull/Netherlands/1/2015(H16N3)
A/black-headed_gull/Netherlands/6/2013(H13N8)
A/black-headed_gull/Netherlands/133/2012(H13N6)
A/black-headed_gull/Netherlands/36/2014(H16N3)
A/black-headed_gull/Republic_of_Georgia/4/2012(H16N3)
A/black-headed_gull/Republic_of_Georgia/9/2011(H13N8)
A/black-headed_gull/Republic_of_Georgia/1/2012(H13N6)
A/yellow-legged_gull/Republic_of_Georgia/5/2012(H13N6)
A/yellow-legged_gull/Republic_of_Georgia/1/2011(H13N6)
A/black-headed_gull/Netherlands/37/2011(H16N3)
A/black-headed_gull/Netherlands/1/2010(H13N6)
A/black-headed_gull/Netherlands/8/2010(H13N2)
A/yellow-legged_gull/Georgia/1/2011(H13N8)
A/black-headed_gull/Netherlands/39/2009(H13N6)
A/mallard/Sweden/101558/2009(mixed)
A/Mongolian_gull/Mongolia/401/2007(H13N6)
A/black_headed_gull/Mongolia/1766/2006(H13N6)
A/black-headed_gull/Netherlands/28/2011(H16N3)
A/black-headed_gull/Sweden/87533/2009(H16N3)
A/black-headed_gull/Netherlands/61/2008(H16N3)
A/black-headed_gull/Netherlands/89/2008(H16N3)
A/glaucous-winged_gull/Southcentral_Alaska/12MB01618/2012(H16N3)
A/Herring_Gull/Delaware/597/2019(H16N3)
A/laughing_gull/Delaware_Bay/453/2018(H16N3)
A/gull/New_Jersey/UGAI15-3414/2015(H16N3)
A/glaucous-winged_gull/Southcentral_Alaska/11JR01736/2011(H16N3)
A/Glaucous-Winged_Gull/Alaska/19MB02459/2019(H16N3)
Laridae
Anatidae
Scolopacidae
Unknown
Charadriidae
Environment
Alcidae
Sulidae
Tree scale
3
2000
2005
2010
2015
2020
2025

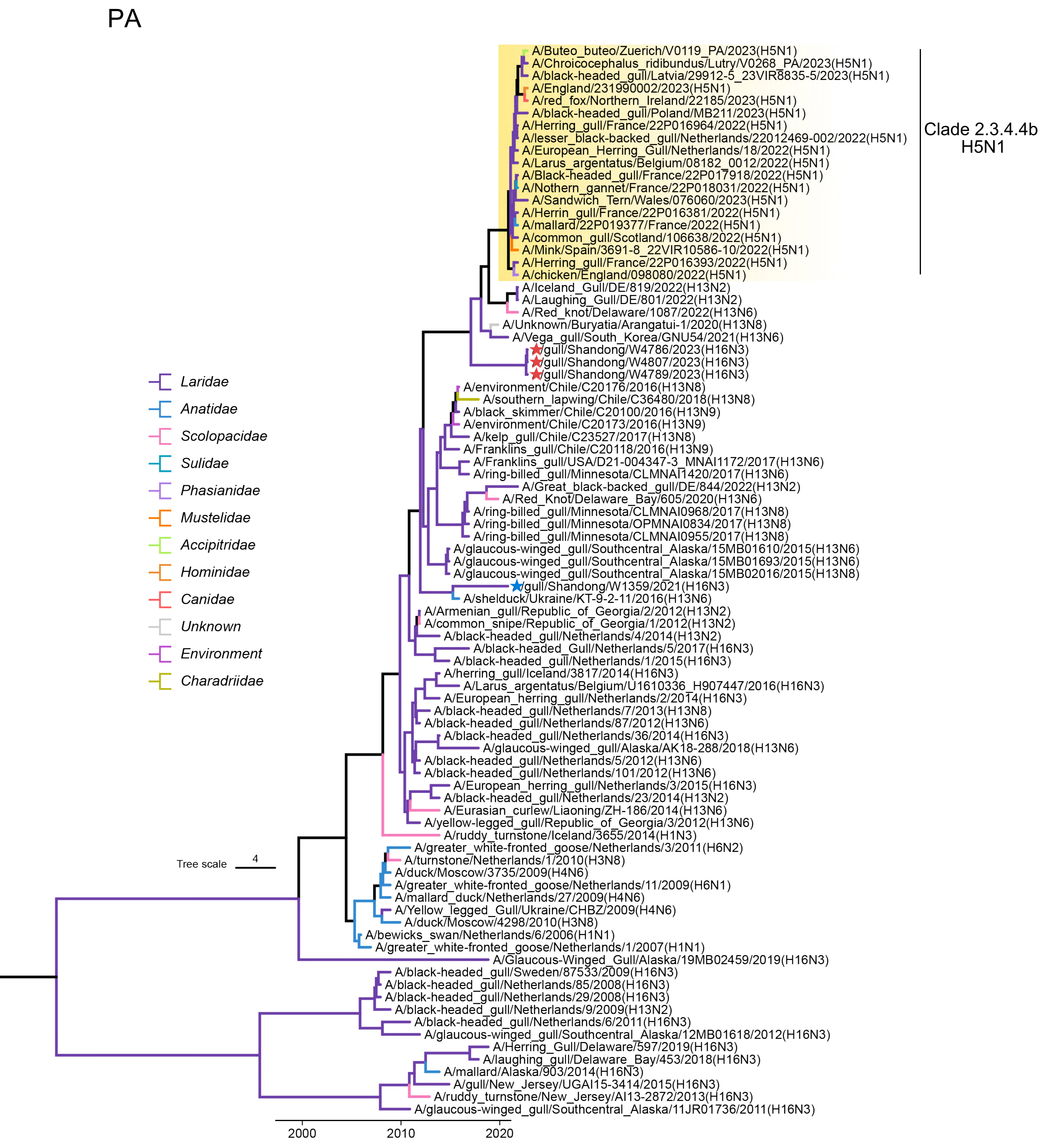
PA
A/Buteo_buteo/Zuerich/V0119_PA/2023(H5N1)
A/Chroicocephalus_ridibundus/Lutry/V0268_PA/2023(H5N1)
A/black-headed_gull/Latvia/29912-5_23VIR8835-5/2023(H5N1)
A/England/231990002/2023(H5N1)
A/red_fox/Northern_Ireland/22185/2023(H5N1)
A/black-headed_gull/Poland/MB211/2023(H5N1)
A/Herring_gull/France/22P016964/2022(H5N1)
A/lesser_black-backed_gull/Netherlands/22012469-002/2022(H5N1)
A/European_Herring_Gull/Netherlands/18/2022(H5N1)
A/Larus_argentatus/Belgium/08182_0012/2022(H5N1)
A/Black-headed_gull/France/22P017918/2022(H5N1)
A/Nothern_gannet/France/22P018031/2022(H5N1)
A/Sandwich_Tern/Wales/076060/2023(H5N1)
A/Herrin_gull/France/22P016381/2022(H5N1)
A/mallard/22P019377/France/2022(H5N1)
A/common_gull/Scotland/106638/2022(H5N1)
A/Mink/Spain/3691-8_22VIR10586-10/2022(H5N1)
A/Herring_gull/France/22P016393/2022(H5N1)
A/chicken/England/098080/2022(H5N1)
Clade 2.3.4.4b
H5N1
A/Iceland_Gull/DE/819/2022(H13N2)
A/Laughing_Gull/DE/801/2022(H13N2)
A/Red_knot/Delaware/1087/2022(H13N6)
A/Unknown/Buryatia/Arangatui-1/2020(H13N8)
A/Vega_gull/South_Korea/GNU54/2021(H13N6)
A/gull/Shandong/W4786/2023(H16N3)
A/gull/Shandong/W4807/2023(H16N3)
A/gull/Shandong/W4789/2023(H16N3)
A/environment/Chile/C20176/2016(H13N8)
A/southern_lapwing/Chile/C36480/2018(H13N8)
A/black_skimmer/Chile/C20100/2016(H13N9)
A/environment/Chile/C20173/2016(H13N9)
A/kelp_gull/Chile/C23527/2017(H13N8)
A/Franklins_gull/Chile/C20118/2016(H13N9)
A/Franklins_gull/USA/D21-004347-3_MNAI1172/2017(H13N6)
A/ring-billed_gull/Minnesota/CLMNAI1420/2017(H13N6)
A/Great_black-backed_gull/DE/844/2022(H13N2)
A/Red_Knot/Delaware_Bay/605/2020(H13N6)
A/ring-billed_gull/Minnesota/CLMNAI0968/2017(H13N8)
A/ring-billed_gull/Minnesota/OPMNAI0834/2017(H13N8)
A/ring-billed_gull/Minnesota/CLMNAI0955/2017(H13N8)
A/glaucous-winged_gull/Southcentral_Alaska/15MB01610/2015(H13N6)
A/glaucous-winged_gull/Southcentral_Alaska/15MB01693/2015(H13N6)
A/glaucous-winged_gull/Southcentral_Alaska/15MB02016/2015(H13N8)
A/gull/Shandong/W1359/2021(H16N3)
A/shelduck/Ukraine/KT-9-2-11/2016(H13N6)
A/Armenian_gull/Republic_of_Georgia/2/2012(H13N2)
A/common_snipe/Republic_of_Georgia/1/2012(H13N2)
A/black-headed_gull/Netherlands/4/2014(H13N2)
A/black-headed_Gull/Netherlands/5/2017(H16N3)
A/black-headed_gull/Netherlands/1/2015(H16N3)
A/herring_gull/Iceland/3817/2014(H16N3)
A/Larus_argentatus/Belgium/U1610336_H907447/2016(H16N3)
A/European_herring_gull/Netherlands/2/2014(H16N3)
A/black-headed_gull/Netherlands/7/2013(H13N8)
A/black-headed_gull/Netherlands/87/2012(H13N6)
A/black-headed_gull/Netherlands/36/2014(H16N3)
A/glaucous-winged_gull/Alaska/AK18-288/2018(H13N6)
A/black-headed_gull/Netherlands/5/2012(H13N6)
A/black-headed_gull/Netherlands/101/2012(H13N6)
A/European_herring_gull/Netherlands/3/2015(H16N3)
A/black-headed_gull/Netherlands/23/2014(H13N2)
A/Eurasian_curlew/Liaoning/ZH-186/2014(H13N6)
A/yellow-legged_gull/Republic_of_Georgia/3/2012(H13N6)
A/ruddy_turnstone/Iceland/3655/2014(H1N3)
A/greater_white-fronted_goose/Netherlands/3/2011(H6N2)
A/turnstone/Netherlands/1/2010(H3N8)
A/duck/Moscow/3735/2009(H4N6)
A/greater_white-fronted_goose/Netherlands/11/2009(H6N1)
A/mallard_duck/Netherlands/27/2009(H4N6)
A/Yellow_legged_Gull/Ukraine/CHBZ/2009(H4N6)
A/duck/Moscow/4298/2010(H3N8)
A/bewicks_swan/Netherlands/6/2006(H1N1)
A/greater_white-fronted_goose/Netherlands/1/2007(H1N1)
A/Glaucous-Winged_Gull/Alaska/19MB02459/2019(H16N3)
A/black-headed_gull/Sweden/87533/2009(H16N3)
A/black-headed_gull/Netherlands/85/2008(H16N3)
A/black-headed_gull/Netherlands/29/2008(H16N3)
A/black-headed_gull/Netherlands/9/2009(H13N2)
A/black-headed_gull/Netherlands/6/2011(H16N3)
A/glaucous-winged_gull/Southcentral_Alaska/12MB01618/2012(H16N3)
A/Herring_Gull/Delaware/597/2019(H16N3)
A/laughing_gull/Delaware_Bay/453/2018(H16N3)
A/mallard/Alaska/903/2014(H16N3)
A/gull/New_Jersey/UGAI15-3414/2015(H16N3)
A/ruddy_turnstone/New_Jersey/AI13-2872/2013(H16N3)
A/glaucous-winged_gull/Southcentral_Alaska/11JR01736/2011(H16N3)
Laridae
Anatidae
Scolopacidae
Sulidae
Phasianidae
Mustelidae
Accipitridae
Hominidae
Canidae
Unknown
Environment
Charadriidae
Tree scale
4
2000
2010
2020

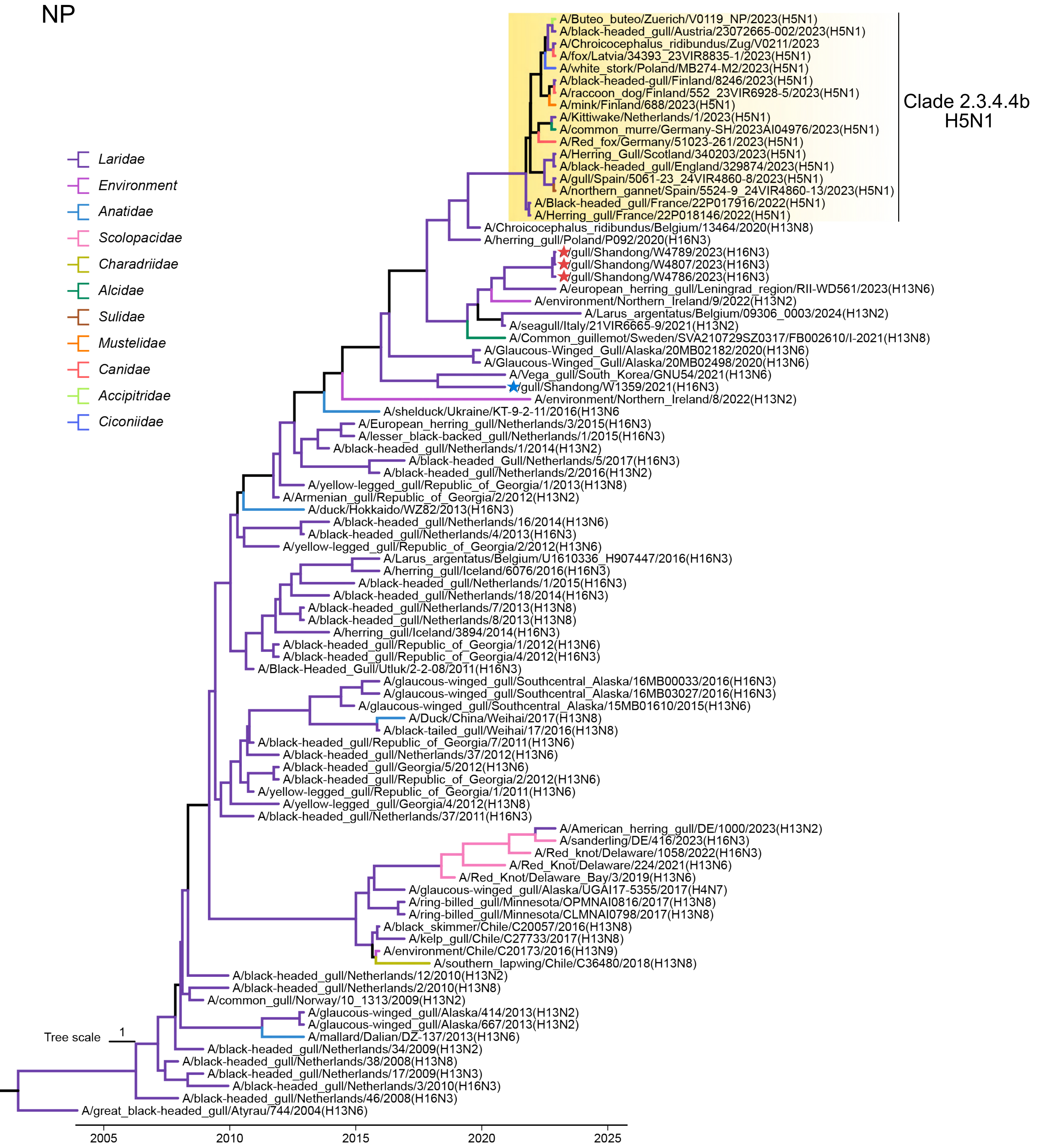
NP
Laridae
Environment
Anatidae
Scolopacidae
Charadriidae
Alcidae
Sulidae
Mustelidae
Canidae
Accipitridae
Ciconiidae
Clade 2.3.4.4b
H5N1
A/Buteo_buteo/Zuerich/V0119_NP/2023(H5N1)
A/black-headed_gull/Austria/23072665-002/2023(H5N1)
A/Chroicocephalus_ridibundus/Zug/V0211/2023
A/fox/Latvia/34393_23VIR8835-1/2023(H5N1)
A/white_stork/Poland/MB274-M2/2023(H5N1)
A/black-headed-gull/Finland/8246/2023(H5N1)
A/raccoon_dog/Finland/552_23VIR6928-5/2023(H5N1)
A/mink/Finland/688/2023(H5N1)
A/Kittiwake/Netherlands/1/2023(H5N1)
A/common_murre/Germany-SH/2023AI04976/2023(H5N1)
A/Red_fox/Germany/51023-261/2023(H5N1)
A/Herring_Gull/Scotland/340203/2023(H5N1)
A/black-headed_gull/England/329874/2023(H5N1)
A/gull/Spain/5061-23_24VIR4860-8/2023(H5N1)
A/northern_gannet/Spain/5524-9_24VIR4860-13/2023(H5N1)
A/Black-headed_gull/France/22P017916/2022(H5N1)
A/Herring_gull/France/22P018146/2022(H5N1)
A/Chroicocephalus_ridibundus/Belgium/13464/2020(H13N8)
A/herring_gull/Poland/P092/2020(H16N3)
A/gull/Shandong/W4789/2023(H16N3)
A/gull/Shandong/W4807/2023(H16N3)
A/gull/Shandong/W4786/2023(H16N3)
A/european_herring_gull/Leningrad_region/RII-WD561/2023(H13N6)
A/environment/Northern_Ireland/9/2022(H13N2)
A/Larus_argentatus/Belgium/09306_0003/2024(H13N2)
A/seagull/Italy/21VIR6665-9/2021(H13N2)
A/Common_guillemot/Sweden/SVA210729SZ0317/FB002610/I-2021(H13N8)
A/Glaucous-Winged_Gull/Alaska/20MB02182/2020(H13N6)
A/Glaucous-Winged_Gull/Alaska/20MB02498/2020(H13N6)
A/Vega_gull/South_Korea/GNU54/2021(H13N6)
A/gull/Shandong/W1359/2021(H16N3)
A/environment/Northern_Ireland/8/2022(H13N2)
A/shelduck/Ukraine/KT-9-2-11/2016(H13N6
A/European_herring_gull/Netherlands/3/2015(H16N3)
A/lesser_black-backed_gull/Netherlands/1/2015(H16N3)
A/black-headed_gull/Netherlands/1/2014(H13N2)
A/black-headed_Gull/Netherlands/5/2017(H16N3)
A/black-headed_gull/Netherlands/2/2016(H13N2)
A/yellow-legged_gull/Republic_of_Georgia/1/2013(H13N8)
A/Armenian_gull/Republic_of_Georgia/2/2012(H13N2)
A/duck/Hokkaido/WZ82/2013(H16N3)
A/black-headed_gull/Netherlands/16/2014(H13N6)
A/black-headed_gull/Netherlands/4/2013(H16N3)
A/yellow-legged_gull/Republic_of_Georgia/2/2012(H13N6)
A/Larus_argentatus/Belgium/U1610336_H907447/2016(H16N3)
A/herring_gull/Iceland/6076/2016(H16N3)
A/black-headed_gull/Netherlands/1/2015(H16N3)
A/black-headed_gull/Netherlands/18/2014(H16N3)
A/black-headed_gull/Netherlands/7/2013(H13N8)
A/black-headed_gull/Netherlands/8/2013(H13N8)
A/herring_gull/Iceland/3894/2014(H16N3)
A/black-headed_gull/Republic_of_Georgia/1/2012(H13N6)
A/black-headed_gull/Republic_of_Georgia/4/2012(H16N3)
A/Black-Headed_Gull/Utluk/2-2-08/2011(H16N3)
A/glaucous-winged_gull/Southcentral_Alaska/16MB00033/2016(H16N3)
A/glaucous-winged_gull/Southcentral_Alaska/16MB03027/2016(H16N3)
A/glaucous-winged_gull/Southcentral_Alaska/15MB01610/2015(H13N6)
A/Duck/China/Weihai/2017(H13N8)
A/black-tailed_gull/Weihai/17/2016(H13N8)
A/black-headed_gull/Republic_of_Georgia/7/2011(H13N6)
A/black-headed_gull/Netherlands/37/2012(H13N6)
A/black-headed_gull/Georgia/5/2012(H13N6)
A/black-headed_gull/Republic_of_Georgia/2/2012(H13N6)
A/yellow-legged_gull/Republic_of_Georgia/1/2011(H13N6)
A/yellow-legged_gull/Georgia/4/2012(H13N8)
A/black-headed_gull/Netherlands/37/2011(H16N3)
A/American_herring_gull/DE/1000/2023(H13N2)
A/sanderling/DE/416/2023(H16N3)
A/Red_knot/Delaware/1058/2022(H16N3)
A/Red_Knot/Delaware/224/2021(H13N6)
A/Red_Knot/Delaware_Bay/3/2019(H13N6)
A/glaucous-winged_gull/Alaska/UGAI17-5355/2017(H4N7)
A/ring-billed_gull/Minnesota/OPMNAI0816/2017(H13N8)
A/ring-billed_gull/Minnesota/CLMNAI0798/2017(H13N8)
A/black_skimmer/Chile/C20057/2016(H13N8)
A/kelp_gull/Chile/C27733/2017(H13N8)
A/environment/Chile/C20173/2016(H13N9)
A/southern_lapwing/Chile/C36480/2018(H13N8)
A/black-headed_gull/Netherlands/12/2010(H13N2)
A/black-headed_gull/Netherlands/2/2010(H13N8)
A/common_gull/Norway/10_1313/2009(H13N2)
A/glaucous-winged_gull/Alaska/414/2013(H13N2)
A/glaucous-winged_gull/Alaska/667/2013(H13N2)
A/mallard/Dalian/DZ-137/2013(H13N6)
A/black-headed_gull/Netherlands/34/2009(H13N2)
A/black-headed_gull/Netherlands/38/2008(H13N8)
A/black-headed_gull/Netherlands/17/2009(H13N3)
A/black-headed_gull/Netherlands/3/2010(H16N3)
A/black-headed_gull/Netherlands/46/2008(H16N3)
A/great_black-headed_gull/Atyrau/744/2004(H13N6)
Tree scale
1
2005
2010
2015
2020
2025

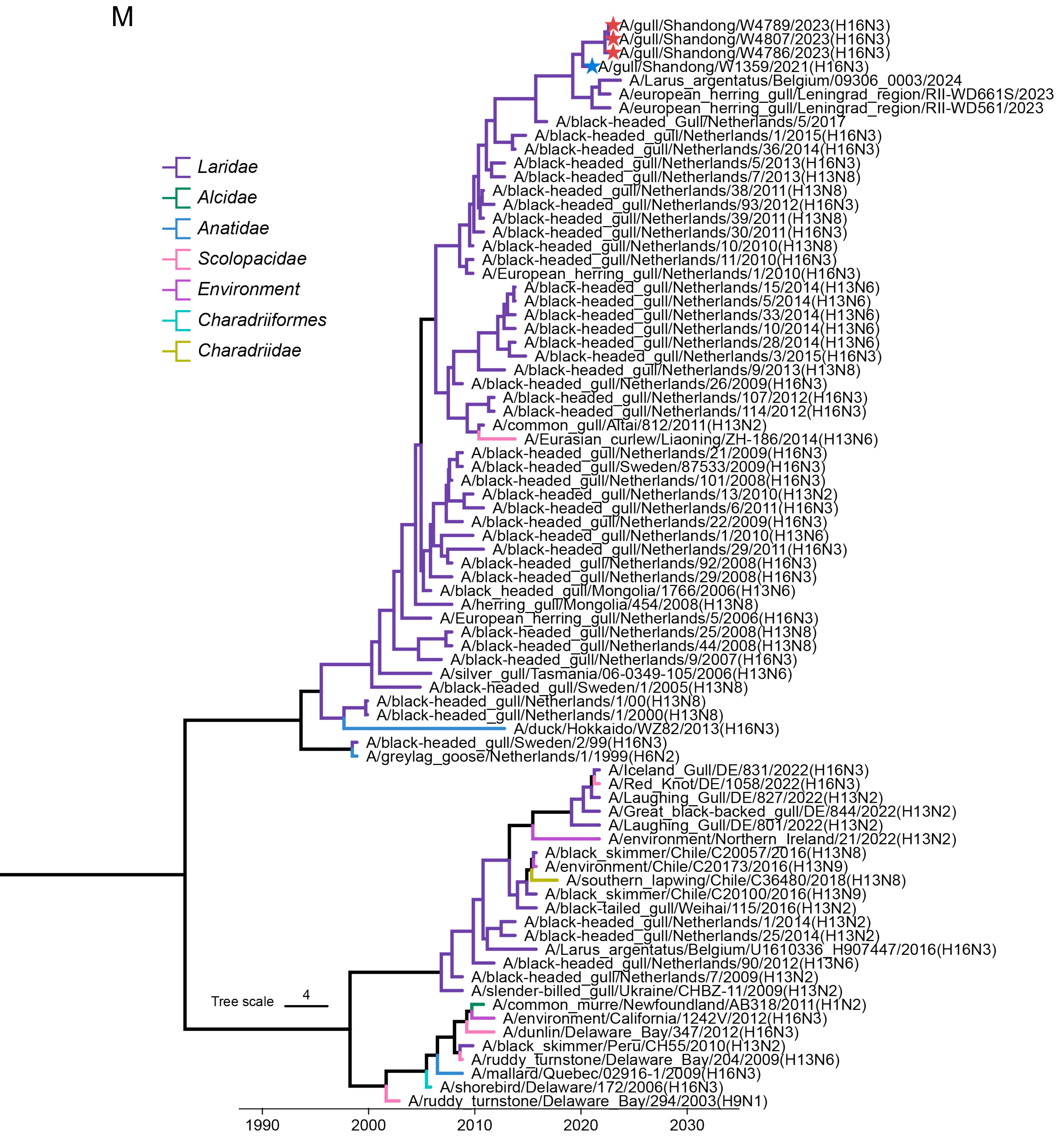
M
Laridae
Alcidae
Anatidae
Scolopacidae
Environment
Charadriiformes
Charadriidae
A/gull/Shandong/W4789/2023(H16N3)
A/gull/Shandong/W4807/2023(H16N3)
A/gull/Shandong/W4786/2023(H16N3)
A/gull/Shandong/W1359/2021(H16N3)
A/Larus_argentatus/Belgium/09306_0003/2024
A/european_herring_gull/Leningrad_region/RII-WD661S/2023
A/european_herring_gull/Leningrad_region/RII-WD561/2023
A/black-headed_Gull/Netherlands/5/2017
A/black-headed_gull/Netherlands/1/2015(H16N3)
A/black-headed_gull/Netherlands/36/2014(H16N3)
A/black-headed_gull/Netherlands/5/2013(H16N3)
A/black-headed_gull/Netherlands/7/2013(H13N8)
A/black-headed_gull/Netherlands/38/2011(H13N8)
A/black-headed_gull/Netherlands/93/2012(H16N3)
A/black-headed_gull/Netherlands/39/2011(H13N8)
A/black-headed_gull/Netherlands/30/2011(H16N3)
A/black-headed_gull/Netherlands/10/2010(H13N8)
A/black-headed_gull/Netherlands/11/2010(H16N3)
A/European_herring_gull/Netherlands/1/2010(H16N3)
A/black-headed_gull/Netherlands/15/2014(H13N6)
A/black-headed_gull/Netherlands/5/2014(H13N6)
A/black-headed_gull/Netherlands/33/2014(H13N6)
A/black-headed_gull/Netherlands/10/2014(H13N6)
A/black-headed_gull/Netherlands/28/2014(H13N6)
A/black-headed_gull/Netherlands/3/2015(H16N3)
A/black-headed_gull/Netherlands/9/2013(H13N8)
A/black-headed_gull/Netherlands/26/2009(H16N3)
A/black-headed_gull/Netherlands/107/2012(H16N3)
A/black-headed_gull/Netherlands/114/2012(H16N3)
A/common_gull/Altai/812/2011(H13N2)
A/Eurasian_curlew/Liaoning/ZH-186/2014(H13N6)
A/black-headed_gull/Netherlands/21/2009(H16N3)
A/black-headed_gull/Sweden/87533/2009(H16N3)
A/black-headed_gull/Netherlands/101/2008(H16N3)
A/black-headed_gull/Netherlands/13/2010(H13N2)
A/black-headed_gull/Netherlands/6/2011(H16N3)
A/black-headed_gull/Netherlands/22/2009(H16N3)
A/black-headed_gull/Netherlands/1/2010(H13N6)
A/black-headed_gull/Netherlands/29/2011(H16N3)
A/black-headed_gull/Netherlands/92/2008(H16N3)
A/black-headed_gull/Netherlands/29/2008(H16N3)
A/black_headed_gull/Mongolia/1766/2006(H13N6)
A/herring_gull/Mongolia/454/2008(H13N8)
A/European_herring_gull/Netherlands/5/2006(H16N3)
A/black-headed_gull/Netherlands/25/2008(H13N8)
A/black-headed_gull/Netherlands/44/2008(H13N8)
A/black-headed_gull/Netherlands/9/2007(H16N3)
A/silver_gull/Tasmania/06-0349-105/2006(H13N6)
A/black-headed_gull/Sweden/1/2005(H13N8)
A/black-headed_gull/Netherlands/1/00(H13N8)
A/black-headed_gull/Netherlands/1/2000(H13N8)
A/duck/Hokkaido/WZ82/2013(H16N3)
A/black-headed_gull/Sweden/2/99(H16N3)
A/greylag_goose/Netherlands/1/1999(H6N2)
A/Iceland_Gull/DE/831/2022(H16N3)
A/Red_Knot/DE/1058/2022(H16N3)
A/Laughing_Gull/DE/827/2022(H13N2)
A/Great_black-backed_gull/DE/844/2022(H13N2)
A/Laughing_Gull/DE/801/2022(H13N2)
A/environment/Northern_Ireland/21/2022(H13N2)
A/black_skimmer/Chile/C20057/2016(H13N8)
A/environment/Chile/C20173/2016(H13N9)
A/southern_lapwing/Chile/C36480/2018(H13N8)
A/black_skimmer/Chile/C20100/2016(H13N9)
A/black-tailed_gull/Weihai/115/2016(H13N2)
A/black-headed_gull/Netherlands/1/2014(H13N2)
A/black-headed_gull/Netherlands/25/2014(H13N2)
A/Larus_argentatus/Belgium/U1610336_H907447/2016(H16N3)
A/black-headed_gull/Netherlands/90/2012(H13N6)
A/black-headed_gull/Netherlands/7/2009(H13N2)
A/slender-billed_gull/Ukraine/CHBZ-11/2009(H13N2)
A/common_murre/Newfoundland/AB318/2011(H1N2)
A/environment/California/1242V/2012(H16N3)
A/dunlin/Delaware_Bay/347/2012(H16N3)
A/black_skimmer/Peru/CH55/2010(H13N2)
A/ruddy_turnstone/Delaware_Bay/204/2009(H13N6)
A/mallard/Quebec/02916-1/2009(H16N3)
A/shorebird/Delaware/172/2006(H16N3)
A/ruddy_turnstone/Delaware_Bay/294/2003(H9N1)
Tree scale
4
1990
2000
2010
2020
2030

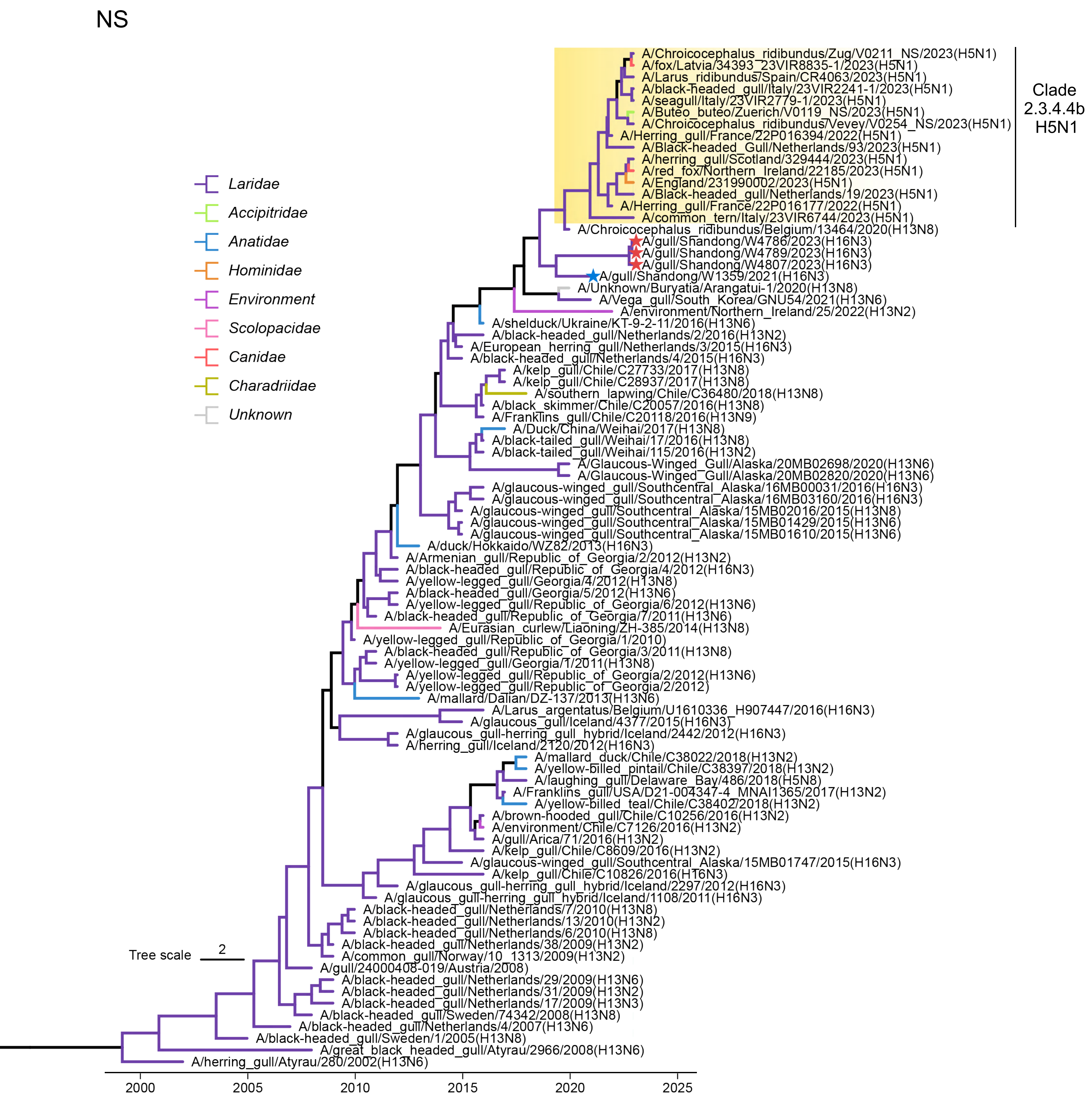
NS
Clade 2.3.4.4b H5N1
Laridae
Accipitridae
Anatidae
Hominidae
Environment
Scolopacidae
Canidae
Charadriidae
Unknown
A/Chroicocephalus_ridibundus/Zug/V0211_NS/2023(H5N1)
A/fox/Latvia/34393_23VIR8835-1/2023(H5N1)
A/Larus_ridibundus/Spain/CR4063/2023(H5N1)
A/black-headed_gull/Italy/23VIR2241-1/2023(H5N1)
A/seagull/Italy/23VIR2779-1/2023(H5N1)
A/Buteo_buteo/Zuerich/V0119_NS/2023(H5N1)
A/Chroicocephalus_ridibundus/Vevey/V0254_NS/2023(H5N1)
A/Herring_gull/France/22P016394/2022(H5N1)
A/Black-headed_Gull/Netherlands/93/2023(H5N1)
A/herring_gull/Scotland/329444/2023(H5N1)
A/red_fox/Northern_Ireland/22185/2023(H5N1)
A/England/231990002/2023(H5N1)
A/Black-headed_gull/Netherlands/19/2023(H5N1)
A/Herring_gull/France/22P016177/2022(H5N1)
A/common_tern/Italy/23VIR6744/2023(H5N1)
A/Chroicocephalus_ridibundus/Belgium/13464/2020(H13N8)
A/gull/Shandong/W4786/2023(H16N3)
A/gull/Shandong/W4789/2023(H16N3)
A/gull/Shandong/W4807/2023(H16N3)
A/gull/Shandong/W1359/2021(H16N3)
A/Unknown/Buryatia/Arangatui-1/2020(H13N8)
A/Vega_gull/South_Korea/GNU54/2021(H13N6)
A/environment/Northern_Ireland/25/2022(H13N2)
A/shelduck/Ukraine/KT-9-2-11/2016(H13N6)
A/black-headed_gull/Netherlands/2/2016(H13N2)
A/European_herring_gull/Netherlands/3/2015(H16N3)
A/black-headed_gull/Netherlands/4/2015(H16N3)
A/kelp_gull/Chile/C27733/2017(H13N8)
A/kelp_gull/Chile/C28937/2017(H13N8)
A/southern_lapwing/Chile/C36480/2018(H13N8)
A/black_skimmer/Chile/C20057/2016(H13N8)
A/Franklins_gull/Chile/C20118/2016(H13N9)
A/Duck/China/Weihai/2017(H13N8)
A/black-tailed_gull/Weihai/17/2016(H13N8)
A/black-tailed_gull/Weihai/115/2016(H13N2)
A/Glaucous-Winged_Gull/Alaska/20MB02698/2020(H13N6)
A/Glaucous-Winged_Gull/Alaska/20MB02820/2020(H13N6)
A/glaucous-winged_gull/Southcentral_Alaska/16MB00031/2016(H16N3)
A/glaucous-winged_gull/Southcentral_Alaska/16MB03160/2016(H16N3)
A/glaucous-winged_gull/Southcentral_Alaska/15MB02016/2015(H13N8)
A/glaucous-winged_gull/Southcentral_Alaska/15MB01429/2015(H13N6)
A/glaucous-winged_gull/Southcentral_Alaska/15MB01610/2015(H13N6)
A/duck/Hokkaido/WZ82/2013(H16N3)
A/Armenian_gull/Republic_of_Georgia/2/2012(H13N2)
A/black-headed_gull/Republic_of_Georgia/4/2012(H16N3)
A/yellow-legged_gull/Georgia/4/2012(H13N8)
A/black-headed_gull/Georgia/5/2012(H13N6)
A/yellow-legged_gull/Republic_of_Georgia/6/2012(H13N6)
A/black-headed_gull/Republic_of_Georgia/7/2011(H13N6)
A/Eurasian_curlew/Liaoning/ZH-385/2014(H13N8)
A/yellow-legged_gull/Republic_of_Georgia/1/2010)
A/black-headed_gull/Republic_of_Georgia/3/2011(H13N8)
A/yellow-legged_gull/Georgia/1/2011(H13N8)
A/yellow-legged_gull/Republic_of_Georgia/2/2012(H13N6)
A/yellow-legged_gull/Republic_of_Georgia/2/2012)
A/mallard/Dalian/DZ-137/2013(H13N6)
A/Larus_argentatus/Belgium/U1610336_H907447/2016(H16N3)
A/glaucous_gull/Iceland/4377/2015(H16N3)
A/glaucous_gull-herring_gull_hybrid/Iceland/2442/2012(H16N3)
A/herring_gull/Iceland/2120/2012(H16N3)
A/mallard_duck/Chile/C38022/2018(H13N2)
A/yellow-billed_pintail/Chile/C38397/2018(H13N2)
A/laughing_gull/Delaware_Bay/486/2018(H5N8)
A/Franklins_gull/USA/D21-004347-4_MNAI1365/2017(H13N2)
A/yellow-billed_teal/Chile/C38402/2018(H13N2)
A/brown-hooded_gull/Chile/C10256/2016(H13N2)
A/environment/Chile/C7126/2016(H13N2)
A/gull/Arica/71/2016(H13N2)
A/kelp_gull/Chile/C8609/2016(H13N2)
A/glaucous-winged_gull/Southcentral_Alaska/15MB01747/2015(H16N3)
A/kelp_gull/Chile/C10826/2016(H16N3)
A/glaucous_gull-herring_gull_hybrid/Iceland/2297/2012(H16N3)
A/glaucous_gull-herring_gull_hybrid/Iceland/1108/2011(H16N3)
A/black-headed_gull/Netherlands/7/2010(H13N8)
A/black-headed_gull/Netherlands/13/2010(H13N2)
A/black-headed_gull/Netherlands/6/2010(H13N8)
A/black-headed_gull/Netherlands/38/2009(H13N2)
A/common_gull/Norway/10_1313/2009(H13N2)
A/gull/24000408-019/Austria/2008)
A/black-headed_gull/Netherlands/29/2009(H13N6)
A/black-headed_gull/Netherlands/31/2009(H13N2)
A/black-headed_gull/Netherlands/17/2009(H13N3)
A/black-headed_gull/Sweden/74342/2008(H13N8)
A/black-headed_gull/Netherlands/4/2007(H13N6)
A/black-headed_gull/Sweden/1/2005(H13N8)
A/great_black_headed_gull/Atyrau/2966/2008(H13N6)
A/herring_gull/Atyrau/280/2002(H13N6)
Tree scale 2
2000
2005
2010
2015
2020
2025

Figure S5

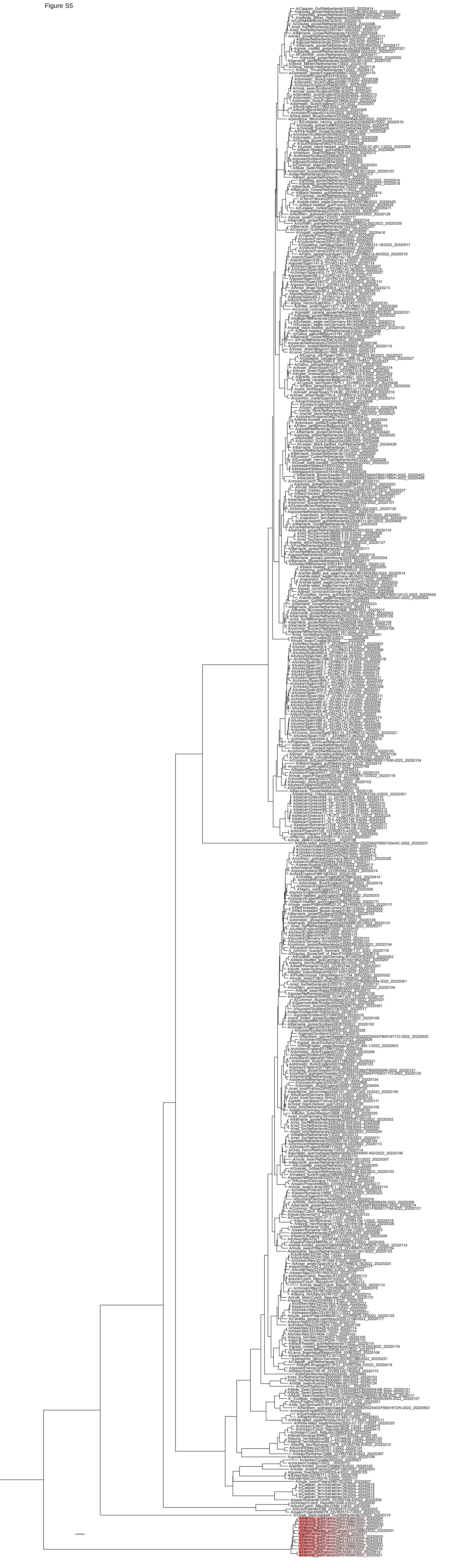

Supplement: Supplementary file 1 [file Data_Sheet_1.pdf]
